# Pressure-driven distillation using air-trapping membranes for fast and selective water purification

**DOI:** 10.1126/sciadv.adg6638

**Published:** 2023-07-14

**Authors:** Duong T. Nguyen, Sangsuk Lee, Kian P. Lopez, Jongho Lee, Anthony P. Straub

**Affiliations:** ^1^ Department of Civil, Environmental, and Architectural Engineering, University of Colorado Boulder, Boulder, CO 80309, USA.; ^2^ Department of Chemical and Biological Engineering, University of Colorado Boulder, Boulder, CO 80309, USA.; ^3^ Department of Civil Engineering, University of British Columbia, Vancouver, British Columbia V6T 1Z4, Canada.; ^4^ Materials Science and Engineering Program, University of Colorado Boulder, Boulder, CO 80309, USA.

## Abstract

Membrane technologies that enable the efficient purification of impaired water sources are needed to address growing water scarcity. However, state-of-the-art engineered membranes are constrained by a universal, deleterious trade-off where membranes with high water permeability lack selectivity. Current membranes also poorly remove low–molecular weight neutral solutes and are vulnerable to degradation from oxidants used in water treatment. We report a water desalination technology that uses applied pressure to drive vapor transport through membranes with an entrapped air layer. Since separation occurs due to a gas-liquid phase change, near-complete rejection of dissolved solutes including sodium chloride, boron, urea, and *N*-nitrosodimethylamine is observed. Membranes fabricated with sub-200-nm-thick air layers showed water permeabilities that exceed those of commercial membranes without sacrificing salt rejection. We also find the air-trapping membranes tolerate exposure to chlorine and ozone oxidants. The results advance our understanding of evaporation behavior and facilitate high-throughput ultraselective separations.

## Teaser

Vapor transport through ultrathin air layers allows for fast, selective, and robust water desalination.

## INTRODUCTION

Anthropogenic climate change and increasing water demands have led to more severe water scarcity, necessitating the use of nontraditional water sources including wastewater, seawater, and brackish water (*1*–*3*). Safe use of these sources requires water treatment systems that remove nearly all dissolved constituents from contaminated water. Membrane technologies, in particular reverse osmosis (RO), have emerged as the premier tools for water reuse and desalination due to their high energy efficiency, ease of operation, and compact design (*4*–*6*).

Despite their widespread implementation, RO systems have experienced longstanding limitations in performance related to membrane materials. Current polymeric salt-rejecting membranes are constrained by a trade-off where high permeability comes at the cost of decreased water-salt selectivity (*7*, *8*). Membranes also routinely fail to remove harmful contaminants since low–molecular weight neutral species can pass through polymer membranes (*9*–*11*); contaminants that are poorly rejected by RO include boron, urea, *N*-nitrosodimethylamine (NDMA), and 1,4-dioxane. Furthermore, the polymeric materials used in RO membranes are vulnerable to chemical oxidation, decreasing longevity, and precluding cleaning of the membranes with chlorine, ozone, and other disinfectants (*12*, *13*).

The fundamental constraints of membranes used in current RO systems motivate the study of alternative separation processes for advanced water treatment. Distillation-based technologies, where separation relies on a gas-liquid phase change, have been used for millennia and maintain key advantages compared to RO. Since separation is accomplished via a phase change, distillation systems remove all low-volatility species from water, including those poorly rejected by RO (*14*–*16*). Distillation technologies can also operate with feedwaters containing harsh oxidants, solvents, and other chemicals (*17*).

The principal drawback of distillation technologies is that they are driven by heat, resulting in high energy consumption associated with thermal losses in the system (*18*, *19*). We therefore pursued the development of a water purification system where pressure, rather than heat, is used to drive a gas-liquid phase transition through a membrane. This pressure-driven distillation process can retain the high energy efficiency and small footprint of RO but also achieve complete removal of nonvolatile species and tolerate harsh feedwaters (*20*). Pressure-driven distillation distinguishes itself from existing thermal membrane distillation since it does not require heat energy and is distinct from osmotic distillation, which requires a secondary separation step (*21*). The feasibility of desalination via pressure-driven distillation is evident from theory, but demonstration of the system has not been possible due to a lack of appropriate membranes for the process (*22*).

**Fig. 1.**
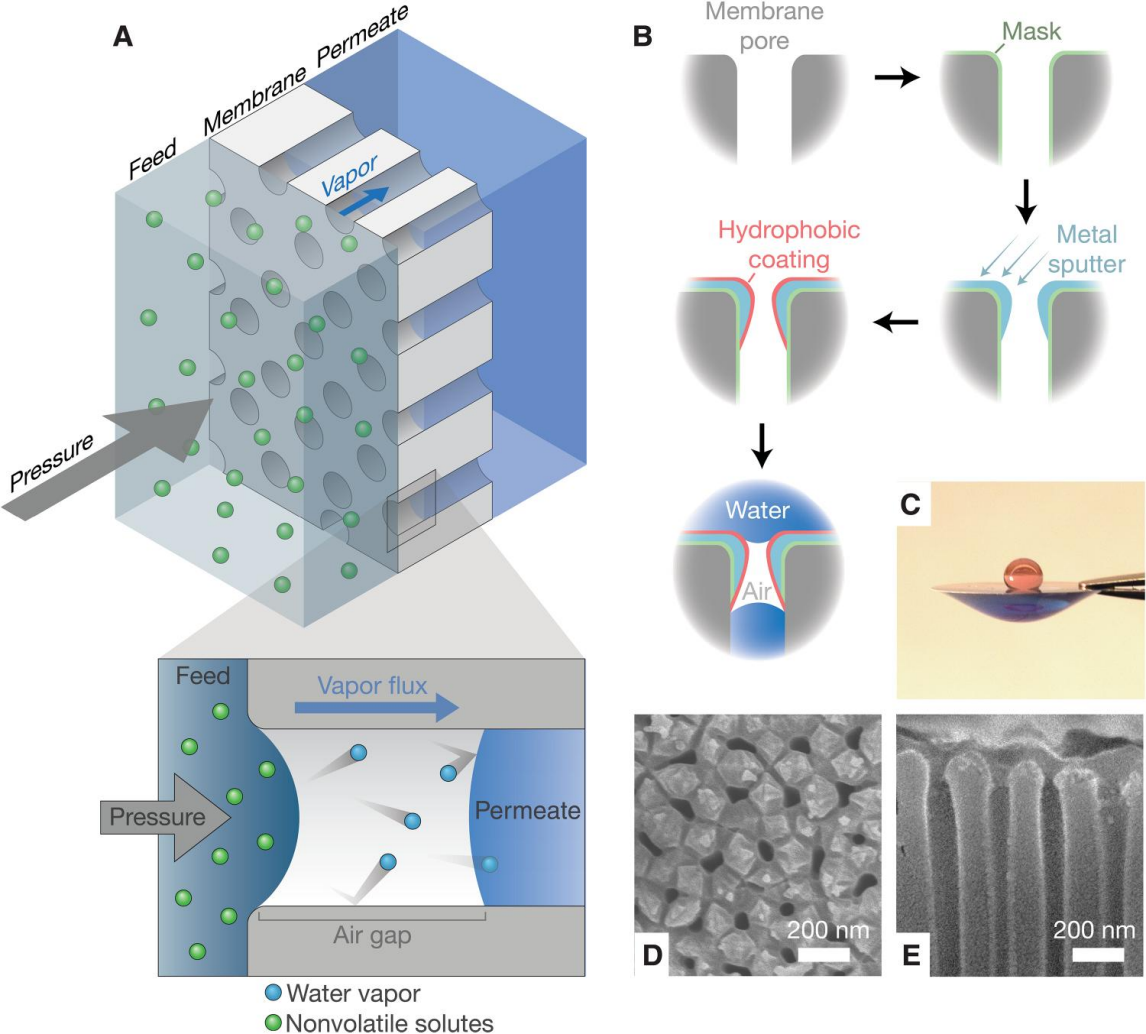
Design of ultrathin air-trapping membranes for pressure-driven vapor transport. (**A**) Schematic diagram of pressure-driven water vapor transport through a nanoporous membrane with an ultrathin air gap. (**B**) Schematic of the fabrication process: A porous alumina membrane is modified with a hydrophilic masking layer, sputtered with controlled metal deposition into pores, selectively coated with a hydrophobic layer on the metal surface, and treated to remove the residual masking layer. (**C**) Water contact angle on the top and bottom surfaces of the membrane. SEM of the (**D**) top surface and (**E**) cross section of the upper surface of the membrane.

In this work, we present a proof-of-concept study on a separation technology that operates using pressure-driven distillation through air-trapping hydrophobic membranes. In this system, applied pressure drives evaporation on the feed side of the membrane, gas-phase diffusion through the pore, and condensation on the permeate side of the membrane (Fig. 1A). Nanoporous membranes with sub-200-nm-thick air layers were synthesized to probe transport and desalination performance. The membranes show near-complete rejection of low-volatility contaminants, including dissolved salts and micropollutants. By decreasing the air layer thickness, high water permeabilities are demonstrated without sacrificing water-salt selectivity, showing the technology can circumvent the permeability-selectivity trade-off that constrains conventional membrane-based desalination systems. The use of an air layer as a separation barrier allows desalination performance to be maintained even when membranes are exposed to chlorine and ozone disinfectants.

## RESULTS

### Pressure-driven distillation through nanoscale air gaps

Air-trapping membranes for proof-of-concept testing were fabricated using porous anodic aluminum oxide (AAO) substrates modified with a controlled hydrophobic coating. The hydrophobic coating was confined to a submicrometer layer on the top surface of the membrane using sequential masking, metal sputtering, and hydrophobic coating with fluorinated alkyl silane (Fig. 1B). The resulting membranes exhibited a superhydrophobic top surface with a water contact angle of 161.2° ± 2.0° and a hydrophilic bottom surface (Fig. 1C). The presence of fluorinated groups associated with the hydrophobic surface coating was confirmed by Fourier transform infrared (FTIR) spectroscopy and energy-dispersive x-ray spectroscopy (figs. S2 and S3). Scanning electron microscopy (SEM) revealed the membranes had uniform pore diameters of 27.1 ± 5.2, 43.3 ± 15.4, or 75.5 ± 14.9 nm depending on the pore size of the initial porous alumina substrate (Fig. 1, D and E, and fig. S4). SEM detection of backscattered electrons confirmed that the hydrophobic metal layer on the top surface of the membrane could be varied between thicknesses of 119.0 ± 12.7 nm and 189.3 ± 34.8 nm based on the sputtering angle (fig. S5). For comparison, membranes with hydrophobic layers spanning the entire membrane thickness (approximately 50 μm) were also fabricated.

**Fig. 2.**
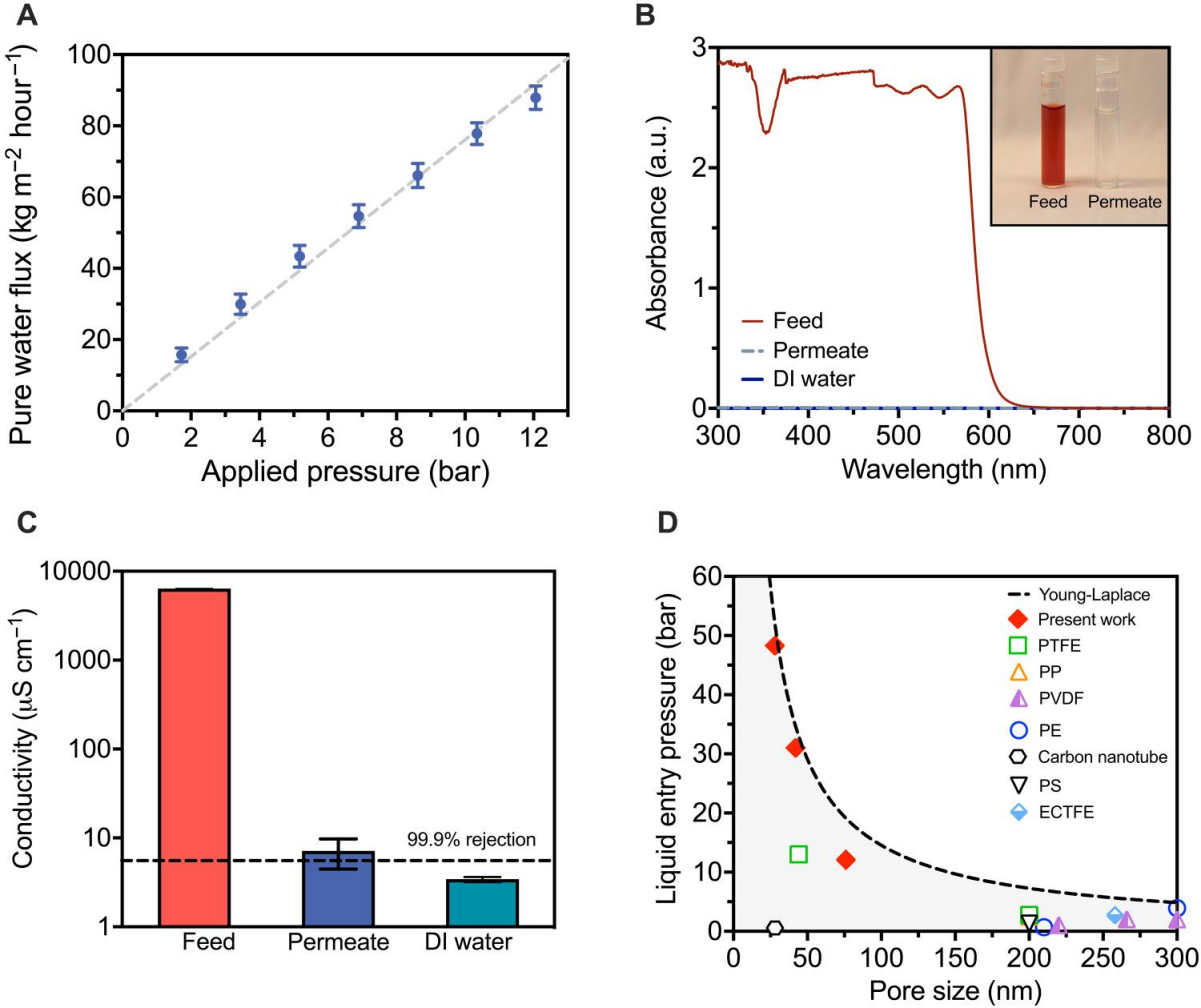
Desalination performance of ultrathin air-trapping membranes. (**A**) Measured water flux as a function of applied hydraulic pressure for 75.5 ± 14.9 nm pore size membranes with a 189-nm-thick hydrophobic layer. Water flux is normalized to the active pore area of the membrane based on a surface porosity of 14.5%. Feed solution was DI water, temperature was 60°C, and hydraulic pressures varied from 1.72 to 12.1 bar. The dashed line is a linear fit to guide the eye. (**B**) UV-Vis spectra for 1 mM Allura Red dye feed water, permeate water, and DI water. The inset photo shows the feed and permeate samples. a.u., arbitrary unit. (**C**) Measured conductivities of the feed, permeate, and DI water for desalination of a 50 mM NaCl solution. The black dashed line depicts 99.9% salt rejection. The operating pressure and temperature were 10.3 bar and 60°C, respectively. (**D**) LEP as a function of pore size for the membranes fabricated in this work and membranes reported in the literature: commercial membranes made of polytetrafluoroethylene (PTFE), polyvinylidene fluoride (PVDF), polypropylene (PP), and polyethylene (PE) and fabricated membranes made of carbon nanotubes, nanofibrous polystyrene (PS), and poly(ethylene chlorotrifluoroethylene) (ECTFE)(*48*–*53*). The dashed curve presents theoretical values obtained from the Young-Laplace equation with an intrinsic contact angle of 120°. In all plots, error bars denote ±1 SD around the mean from three separate membranes.

Air-trapping membranes have not been previously used in pressurized applications because liquid water can enter the pores when small hydraulic pressures (3 to 5 bar) are applied, compromising water-salt selectivity (table S1). We found that the sub-100-nm pore size hydrophobic membranes resist wetting at high hydraulic pressures and demonstrate pressure-driven water vapor flow that increases with increasing hydraulic pressure (Fig. 2A). For 75.5 ± 14.9 nm pore size membrane, the water flux increased monotonically with pressure, reaching pore-area-normalized fluxes up to 88.0 kg m^−2^ hour^−1^ at pressures of 12.1 bar. Removal experiments for allura red AC dye (496 g/mol, Stokes diameter of approximately 1 nm) over 24 hours quantified using ultraviolet-visible (UV-Vis) absorbance showed that permeate samples had identical spectra as deionized (DI) water in the measured wavelength range (300 to 800 nm), indicating more than 99.99% dye removal (Fig. 2B). Salt rejection experiments with a 50 mM sodium chloride feed solution measured for a 24-hour period observed rejections higher than 99.8%, and permeate samples had electrical conductivities approaching those of DI water (Fig. 2C). Long-term experiments over a 7-day period found that salt rejection and water flux rates could be maintained in fabricated membranes (fig. S8). Water flux measurements were consistent with transport models based on water evaporation rates and Maxwell-Stefan diffusion theory. Simulated water fluxes using membrane properties (e.g., pore size, thickness, and porosity) determined via SEM without fitting parameters showed less than 15% deviation from experimental measurements (fig. S7).

Decreasing the pore sizes of the membrane allowed for operation at high hydraulic pressures without pore wetting (Fig. 2D). The liquid entry pressure (LEP) describes the pressure at which the air layer entrapped within the membrane pore is displaced by liquid water. The measured LEPs of membranes with pore sizes of 75.5 ± 14.9, 43.3 ± 15.4, and 27.1 ± 5.2 nm were 12.1 ± 1.7, 20.8 ± 3.5, and 48.3 ± 5.2 bar, respectively. These maximum pressures were aligned with predictions based on the Young-Laplace equation, and we therefore expect that higher operating pressures will be possible by further decreasing the membrane pore size. The LEP of 48.3 ± 5.2 bar measured for the 27.1 ± 5.2 nm pore size membrane was more than a factor of 10 higher than previous air-trapping desalination membranes and appropriate for treating seawater (typical osmotic pressure of 28 bar) (*23*, *24*).

### Achievable water permeability and salt rejection

**Fig. 3.**
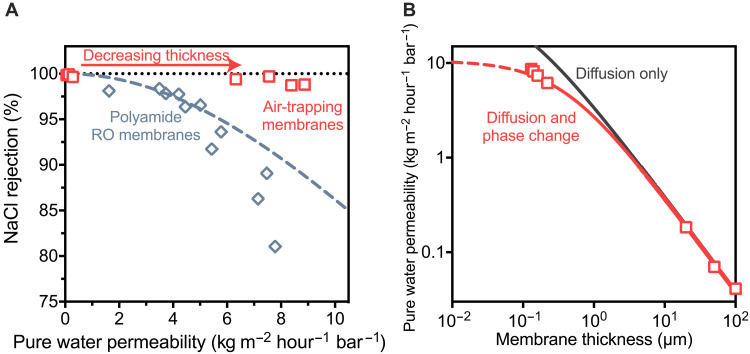
Achievable water permeability in air-trapping membranes. (**A**) Normalized pure water permeability and sodium chloride rejection of air-trapping membranes (red squares). The permeability of air-trapping membranes was increased by decreasing the thickness of the air layer from 100 μm to 119 nm. Permeability values of air-trapping membranes are normalized to the active pore area based on a surface porosity of 14.5%. Membranes were tested with 50 mM NaCl at a temperature of 60°C and an applied pressure of 10.3 bar. For comparison, the permeability-selectivity trade-off curve for polyamide membranes is shown from experimental measurements (blue diamonds) and theoretical estimations (dotted blue line). (**B**) Water permeability of air-trapping membranes as a function of membrane thickness (red squares). Simulated curves based on only diffusion resistances or diffusion and phase-change resistances are shown. Phase-change resistances were modeled using a condensation coefficient of 0.33. Dashed lines indicate thicknesses below the minimum thickness that causes pore wetting.

State-of-the-art membranes are subject to a trade-off between water permeability and water-salt selectivity where a gain in water permeability results in a loss in salt rejection (*25*, *26*). In contrast, we observed membranes that rely on a gas-liquid phase change can increase permeability without sacrificing water-salt selectivity by decreasing the thickness of the air layer. We show shortening the air layer thickness to 119.0 ± 12.7 nm allows for the water permeability of the entrapped air layer (that is, the permeability normalized the active pore area) to reach values up to 8.9 kg m^−2^ hour^−1^ bar^−1^ while maintaining greater than 99% salt rejection (Fig. 3A). Since the membranes have a surface porosity of 14.5%, the water permeability normalized to the total membrane area is 1.3 kg m^−2^ hour^−1^ bar^−1^. The lack of a measurable change in salt rejection as the thickness decreased was consistent with our understanding that selectivity in the air-trapping membranes is attributable to the gas-liquid phase change transport mechanism. Because of their low thicknesses, the water permeabilities of membranes in this work were notably higher than previous work in osmotic and membrane distillation systems, where membranes are tens of micrometers thick and generally have water permeabilities one to two orders of magnitude lower (*21*). While the aim of this study was not to produce membranes that outcompete commercial RO membranes in terms of water permeability, our measurements provide experimental evidence that it is possible for air-trapping membranes to reach the water permeabilities needed for efficient water desalination (*27*).

The high water permeabilities observed in this study are particularly revealing because the fabricated membranes approach the minimum air layer thickness derived from thermodynamic wetting theory (*28*, *29*). Pore wetting is thermodynamically unfavorable when the grand potential of the wetted state is greater than the grand potential of the unwetted air-filled state. As the aspect ratio of the hydrophobic pore decreases (that is, as the air layer becomes thinner), spontaneous pore wetting due to capillary condensation becomes more favorable. For the 75.5-nm-diameter pores studied in this work, the minimum predicted thickness for the air layer is 116.8 nm, assuming an intrinsic water contact angle of 120°. In our testing, water permeability could be measured for membranes with a thickness down to 119.0 nm, and membranes with a thinner hydrophobic layer showed spontaneous pore wetting. Our measurements therefore showed good agreement with theory and provide experimental support for two-decade-old predictions on the minimum thickness of a hydrophobic pore (*28*, *29*). Since the pores here approach the minimum possible aspect ratio, the observed water permeabilities may be near the upper limit possible using a vapor transport through an air-filled pore (*22*).

Latent heat transfer by water molecules traveling through the entrapped air layer was found to have a negligible impact on the water flux. Evaporation on the feed air-liquid interface and condensation on the permeate air-liquid interface result in cooling and heating, respectively, due to latent heat transfer through the membrane. However, conductive heat transfer through the ultrathin air layer and the membrane material transfers heat back to the feed stream, mitigating the buildup of a temperature difference. Therefore, no temperature difference across the membrane was observed in any of our tests, and theoretical simulations showed that the system operates under near isothermal conditions with a temperature difference across the membrane less than 1 × 10^−3^°C (figs. S10 and S11). Since temperature effects in the process are negligible, we expect that the energy efficiency of pressure-driven distillation will be similar to that of RO since both processes are driven by hydraulic pressure and offer comparable water fluxes (*20*).

Decreasing the air layer thickness to less than 200 nm causes transport to occur in a regime where vapor permeability is limited by resistances associated with evaporation and condensation rather than diffusion resistances (*28*). For membranes with high vapor layer thicknesses (20 to 100 μm), we found that transport theory based on only diffusion resistances showed good agreement with measured water permeabilities (Fig. 3B). However, when the vapor layer thickness decreased to below 200 nm, water permeability values were lower than those predicted using only diffusion resistances, indicating substantial contributions from resistances at the liquid-vapor interface. The resistances caused by the phase change are described by the condensation coefficient, σ, a fundamental property which quantifies the probability that an incident vapor molecule condenses at the vapor-liquid interface (*30*). The condensation coefficient has been difficult to probe experimentally, and measured values have varied two orders of magnitude due to major uncertainties related to temperature, interface area, and salinity (*31*). Calculations based on our experimental measurements found that the condensation coefficient at 0.68 bar and 60°C was 0.27 in agreement with a previous study using air-trapping membranes (*30*). Our ability to estimate the condensation coefficient demonstrates the utility of air-trapping membranes as a platform to study gas-liquid phase-change phenomena, and further work may be able to accurately measure this coefficient under a variety of experimental conditions.

### Solute rejection and chemical resilience

**Fig. 4.**
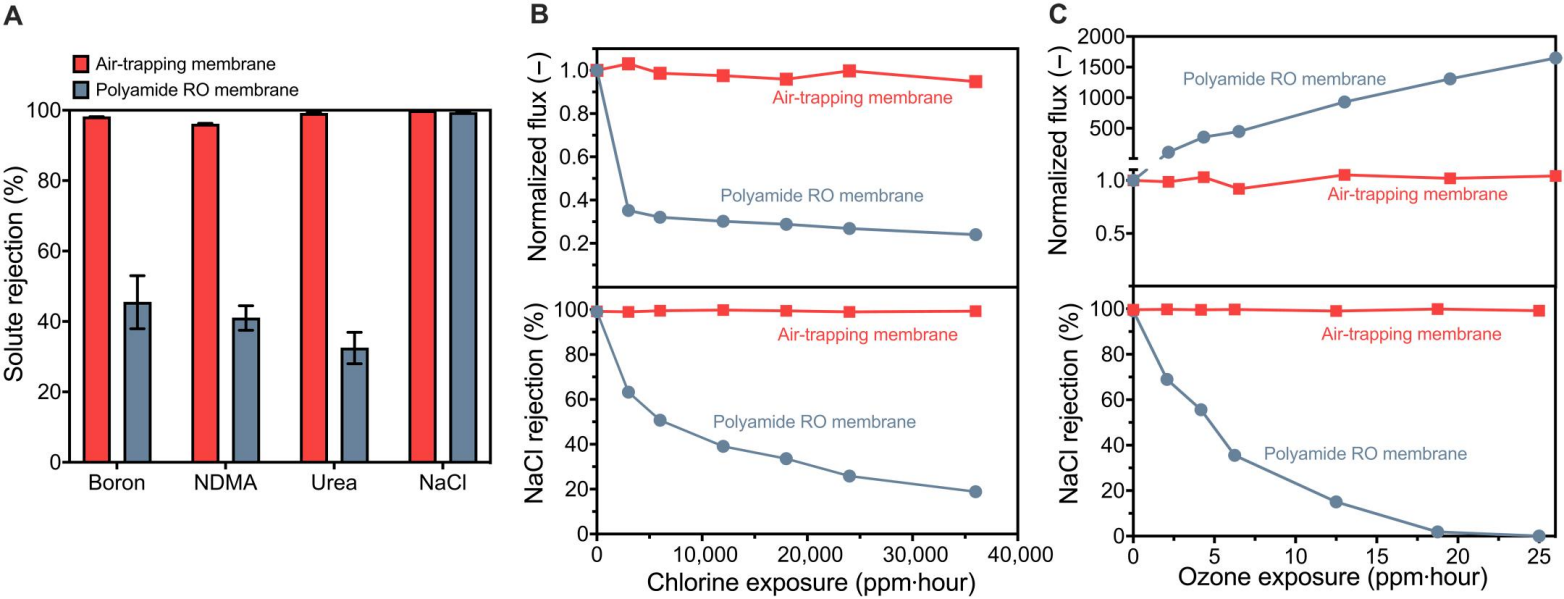
Selectivity and oxidation resistance of fabricated membranes. (**A**) Rejection of boron, NDMA, urea, and sodium chloride by the fabricated air-trapping membranes with a pore size of 75.5 ± 14.9 nm and commercial polyamide RO membranes (Dow SW30). Error bars indicate SD for duplicate experiments using two membranes fabricated in the same procedure. (**B**) Effect of chlorine exposure on the water flux and salt rejection of the fabricated air-trapping membranes and polyamide RO membranes. Chlorine concentration is 1000 ppm at pH 4, and exposure time varies from 1 to 36 hours. (**C**) Effect of ozone exposure on membrane water flux and salt rejection. Ozone concentration is 25 ppm at pH 7, and exposure time varies from 0.083 to 1 hour. The applied hydraulic pressure was fixed at 10.3 bar for all experiments.

Using a vapor layer as a separation barrier results in fundamentally different selective properties as compared to conventional RO membranes that use a thin polymer film. Air-trapping membranes were evaluated for the removal of three contaminants poorly rejected by commercial polyamide RO membranes: boron, urea, and NDMA (*10*, *27*). The solution pH for each contaminant was adjusted to below the pKa to ensure that undissociated species were dominant in the feed water. The air-trapping membranes demonstrated 99.1 and 98.1% rejection of boron and urea, respectively, indicating near-complete removal of both solutes (Fig. 4A). In contrast, polyamide RO membranes poorly rejected boron and urea with 45.5 and 35.6% rejection, respectively. Rejection of NDMA in the fabricated air-trapping membranes was 95.8% due to the slight volatility of NDMA but still exceeded the 43.4% rejection observed with polyamide RO membranes. The high rejections are consistent with our understanding of the air layer acting as a near-impermeable separation barrier for nonvolatile solutes (*16*, *24*, *32*).

Separations with an entrapped air layer enable the use of oxidation-resistant hydrophobic membrane materials. Desalination experiments were conducted with fabricated membranes after exposures to high doses of chlorine and ozone, two strong oxidants used in water treatment processes (Fig. 4, B and C) (*12*). After chlorine exposure of 36,000 parts per million (ppm) hour^−1^ at pH 4 (1000 ppm for 36 hours) or ozone exposure of 25 ppm hour^−1^ at pH 7 (25 ppm for 1 hour), the membranes demonstrated greater than 99% NaCl rejection and less than 6% variation in water flux. Conversely, polyamide RO membranes exposed to chlorine and ozone showed less than 20% salt rejection and severe changes in the water flux. The unchanged structure and chemistry of the fabricated membranes after oxidative exposure were confirmed using SEM, FTIR, and contact angle analysis (figs. S1, S2, and S4).

## DISCUSSION

We have demonstrated a pressure-driven distillation process for water purification that uses applied pressure to drive vapor flow through an air-trapping membrane. Proof-of-concept experiments found that such membranes can achieve high rejection (greater than 99%) of nonvolatile solutes, including sodium chloride, boron, and urea. Membranes with a 27.1-nm pore diameter were found to operate at pressures up to 48.3 bar. By decreasing the thickness of the air layer to 119.0 nm, we find that normalized permeabilities of up 8.9 kg m^−2^ hour^−1^ bar^−1^ can be achieved without sacrificing salt rejection. We also find that the performance of air-trapping membranes is unaffected by exposure to sustained high concentrations of chlorine and ozone.

Since the desalination approach presented here relies on using an applied pressure to drive flow through a semi-permeable membrane, we expect that air-trapping membranes can be substituted directly for conventional RO membranes with the potential advantages of improved permeability, selectivity, and oxidation resistance. In desalination applications, the high selectivity of air-trapping membranes toward nonvolatile solutes may obviate the costly secondary treatment processes currently needed to remove boron since relatively low concentrations of the contaminant (0.5 to 1 mg liter^−1^) can harm agriculture (*33*). For water reuse applications, high removal of NDMA, urea, and other contaminants may improve the safety of product water and reduce the need for further downstream processes (*10*). We also envision the high selectivity of the air-trapping membrane may allow for more efficient ultrapure water production and water recycling in space applications (*34*).

A key advantage of pressure-driven distillation is that it can potentially use a variety of membrane materials and structures to achieve the required air-trapping structure, allowing for more tailored and chemically robust water treatment systems than conventional salt-rejecting membranes that rely on polyamide or cellulose acetate chemistry. Here, we find that fabricated hydrophobic alumina membranes resist damage from chemical oxidants such as chlorine and ozone. We expect that other more scalable materials, including hydrophobic polymers, can be developed for the process and offer similar resistance to chemical oxidation (*6*, *17*, *35*, *36*). The ability to use free chlorine or ozone, rather than chloramines, in treatment trains can prevent the formation of certain harmful disinfection byproducts such as NDMA (*37*). Strong oxidants like chlorine and ozone also inhibit fouling associated with organic and biological matter, providing potentially transformative improvements in water treatment performance and membrane longevity (*12*).

Continued investigations are needed to address challenges of pressure-driven distillation related to wetting, fouling, and scale-up. Air-trapping membranes are vulnerable to wetting from low–surface tension liquids and foulants, and further work is needed to comprehensively identify fouling behavior. Efforts are also required to fabricate scalable large-area hydrophobic porous membranes. Future membranes should have small monodisperse pore sizes to operate at high applied pressure without wetting and use thin air layers to maximize permeability while avoiding trade-offs associated with membrane wetting (*38*). Membranes should also have high surface porosities to maximize the achievable active membrane area. Tailored structures that have already shown promise in related membrane distillation processes, such as omniphobic or Janus designs, may also offer the potential for most robust separations (*39*).

Although this work has laid out the fundamental principles of pressure-driven distillation technology, key knowledge gaps exist related to interfacial and evaporation phenomena that underlie the process. In this study, we fabricated membranes with air layers that approached the minimum thickness possible based on thermodynamic models (*29*). We also experimentally detected interfacial resistances related to molecular reflection at the gas-liquid interface. Continued work is needed to establish a complete theoretical framework for transport in air-filled nanopores and identify the importance of interfacial resistances, nanoconfinement, cluster evaporation, and other behaviors that have been hypothesized in the literature (*40*, *41*). We therefore anticipate that the membranes in this work can serve as a platform to study broadly important interfacial and evaporation phenomena.

## MATERIALS AND METHODS

### Fabrication of membranes with an ultrathin hydrophobic layer

Membranes with an ultrathin hydrophobic layer were fabricated using flat-sheet AAO membranes with varying pore diameters (27.1, 43.3, and 75.5 nm), a thickness of 50 μm, and a macroscopic diameter of 13 mm as the substrates (InRedox, CO, USA). The substrates were sandwiched between polished alumina plates to prevent deformation and annealed at 1000°C for 2 hours. The polycrystalline membranes were then exposed to the vapor of 3-aminopropyl triethoxysilane (Sigma-Aldrich, MO, USA) at a temperature of 70°C and a vacuum pressure of 20 MPa for 4 hours to create a hydrophilic layer of amine groups that would mask subsequent silane-based surface modification. Platinum was then deposited on the membranes using a magnetron sputter coater (Leica ACE600, Germany) with varying incident angles of 55° to 75° using argon as sputtering gas under a target-substrate distance 50 mm and a current of 35 mA. The incident angle is the angle at which the sputter approaches the membrane, an incident angle of 90° would be perpendicular to the membrane surface. The penetration depth inside alumina nanopores was tailored using geometric calculations verified with imaging and experimental measurements. Platinum-deposited membranes were then exposed to a hydrophobic grafting chemical, (heptadecafluoro-1,1,2,2-tetrahydrodecyl) triethoxysilane (Gelest Inc., PA, USA) at 90°C for 12 hours with a vacuum pressure of 20 MPa to selectively functionalize the platinum layer. The resulting membranes were floated on the interface of a mixture of 20% tetrabutylammonium fluoride/water (Thermo Fisher Scientific, MA, USA) for 30 min to remove the hydrophilic aminosilane layer. The membranes were lastly rinsed with ultrapure water several times, dried with nitrogen gas, and placed under vacuum at 120°C for 2 hours.

Membranes with a thick hydrophobic layer were fabricated by coating the entire thickness of AAO with hydrophobic chemicals. These thicker membranes were used in some desalination testing and to validate models for transport. AAO membranes were annealed at 1000°C for 2 hours and then immersed in 30% H_2_O_2_ solution (Thermo Fisher Scientific, MA, USA) at 120°C for 30 min and thoroughly dried at 120°C for 1 hour and under nitrogen gas. (heptadecafluoro-1,1,2,2-tetrahydrodecyl) triethoxysilane was grafted on the resulting membranes via vapor deposition in a vacuum oven at 120°C for 3 hours under a vacuum pressure of 20 MPa. The modified membranes were rinsed with ultrapure water, dried with nitrogen gas, and heated at 120°C for 2 hours.

### Material characterizations of the fabricated membranes

Membrane hydrophobicity was evaluated via water contact angle measurements with a calibrated tensiometer (Biolin Scientific, AZ, USA) using the sessile drop method with one water droplet of 20 μl. The top surface and cross section of the membranes were imaged by field-emission SEM (FESEM) using secondary and backscattered electron detectors (JEOL, Tokyo, Japan). Membrane samples were coated with 5 nm of carbon thread before taking images. The average pore size, porosity, and thickness of the membranes were extracted from FESEM images at various locations using ImageJ software (National Institute of Health, MD, USA). Five SEM images from similarly fabricated alumina membranes were used for ImageJ analysis and the surface pore size was considered to be the longest distance between two points on the pore boundaries (or the Feret’s diameter). Elemental compositions of the membranes were quantified using an energy-dispersive x-ray spectroscopy detector (Oxford Instruments, Oxfordshire, UK) coupled with the FESEM system operating at an accelerating voltage of 15 kV. FTIR was conducted using a spectrometer with a diamond attenuated total reflection module (Cary 630, CO, USA).

### Measurement of water flux and solute rejection

Transport characterization was conducted using a membrane flow cell immersed in a DI water bath with regulating temperature and varying hydraulic pressure applied using a nitrogen gas cylinder (fig. S12). The test cell and adjacent tubing were immersed in a DI water bath with regulated temperature. Before each experiment, the test cell, feed channel, and permeate channel were cleaned by ultrapure water several times until less than 1 μS cm^−1^ conductivity was detected when passing DI water through the feed and permeate tubes. The membranes with a 13 mm diameter were placed inside the cell and tightly sealed with fitted rubber O-rings. The feed channel was filled with water containing a solute (NaCl, boron, urea, or NDMA) and the permeate channel was filled with DI water to enable the condensation of water vapor. Measurements were only recorded when the pressure and thermal equilibrium of the system had been reached after 4 hours.

Water flux was determined by precisely monitoring the volume of water gained in the permeate tube and confirmed with the loss in volume of the feed. A digital camera with high resolution was set up to acquire images of the feed and permeate tubes to monitor water level changes as a function of time (Logitech C390s, CA, USA). NaCl concentration was measured using a calibrated conductivity meter (Oakton CON2700, MA, USA) and corrected for dilution in the permeate tube. Allura dye concentration was analyzed via its absorbance at 500 nm by a UV-Vis spectrophotometer (Hach DR6000, CO, USA). Urea concentration was quantified using a colorimetric method of urea and diacetyl monoxime, allowing the detection limit down to 100 nM (*42*). Boron concentration was analyzed by inductively coupled plasma mass spectroscopy with a detection limit of 0.24 μg/liter (*43*). NDMA concentration was measured by high-performance liquid chromatography equipped with a C18 column using a mobile phase of ultrapure water/methanol with a ratio of 90:10 running at a flow rate of 1 ml min^−1^ (*44*).

### Theoretical modeling of membrane performance

The transport of a multicomponent gas mixture (water vapor and air) through a porous medium was simulated using the Dusty-Gas Model, an extension of the Stefan-Maxwell diffusion theory. This model has been experimentally corroborated in binary and ternary gas systems for the diffusion of inert gases under uniform pressure from the Knudsen to molecular diffusion regime and thus is applicable to model our system (*28*, *45*). A recent numerical study has found that the dusty-gas model is valid even for very low aspect ratio pores such as those used in this study (*46*).

Transport of water across a pore can be described by relating the vapor pressure to the maximum theoretical mass flux from an interface, *S,* by the Hertz hypothesis (*47*)

$$S=\sqrt{\frac{M}{2\pi R_{g}}}\frac{P_{v}}{\sqrt{T}}$$ (1)

where *M* is molar mass, *R*_g_ is the universal gas constant, *P*_v_ is partial vapor pressure, and *T* is temperature. This expression can be used to find the difference in evaporation rates at both ends of the pore, ∆*S*, and can be further simplified by assuming an isothermal condition across the air gap

$$\text{Δ}S=\sqrt{\frac{M}{2\pi R_{g}}}\left(\frac{P_{v,f}}{\sqrt{T_{f}}}-\frac{P_{v,p}}{\sqrt{T_{p}}}\right)≅\sqrt{\frac{M}{2\pi R_{g}T}}\text{Δ}P_{v}$$ (2)

where *P*_v,f_ and *P*_v,p_ are the partial vapor pressures at the feed and permeate sides of the pore, respectively, and *T*_f_ and *T*_p_ are the temperatures at the feed and permeate sides of the pores, respectively. Water mass flux across the membrane (*J*_w_) can then be expressed as the product of membrane porosity, ε, and the difference in evaporation rate, divided by the total resistances experienced as water traverses the pore

$$J_{w}=\varepsilon \frac{\text{Δ}S}{\sum R}≅\varepsilon \sqrt{\frac{M}{2\pi R_{g}T}}{\left(R_{t}+R_{i,f}+R_{i,p}\right)}^{-1}\text{Δ}P_{v}=B_{w}\text{Δ}P_{v}$$ (3)

∑*R* is the sum of multiple mass transport resistances where *R*_t_ corresponds to the transmission resistances, and *R*_i,f_ and *R*_i,p_ correspond to the interfacial resistances that occur on the feed and permeate sides of the membrane, respectively. *B*_w_ is the vapor permeability coefficient, a proportionality factor which relates flux to a partial vapor pressure difference including contributions from both transmission and interfacial resistances.

The water flux defined by Eq. 3 is directly proportional to the vapor pressure difference across the membrane. From Raoult’s law and the Kelvin equation, the water vapor pressure as a function of temperature concentration, and hydraulic pressure can be expressed as

$$P_{v}(T,C,P_{h})=P_{v,0}(T)a_{w}(C)\exp\left(\frac{P_{h}V_{m}}{R_{g}T}\right)=P_{v,0}(T)\exp\left[\frac{(P_{h}-\pi )V_{m}}{R_{g}T}\right]$$ (4)

where *P*_h_ is the hydraulic pressure relative to the ambient pressure, and *V*_m_ is the molar volume of liquid water. The water activity, *a*_w_(*C*), can be related to the osmotic pressure as follows $\pi =-\frac{R_{g}T}{V_{m}}\ln a_{w}(C)$ to obtain the second expression in Eq. 4. The equilibrium vapor pressure as a function of temperature, *P*_v,0_(*T*), can be calculated using the Antoine equation

$$P_{v,0}(T)=10^{A-\frac{B}{C+T}}$$ (5)

where *A*, *B*, and *C* are the Antoine constants for water.

Combining Eqs. 3 and 4[Disp-formula E5], the overall water mass flux across the membrane can be expressed as follows

$$J_{w}=B_{w}[P_{v}(T_{f,m},C_{f,m},P_{h,f})-P_{v}(T_{p,m},C_{p,m},0)]≅B_{w}\frac{P_{v,0}(T)V_{m}}{R_{g}T}(\text{Δ}P-\text{Δ}\pi )$$ (6)

where *T*_f_,_m_ and *T*_p,m_ are temperatures at the membrane surface on the feed and permeate, respectively; *C*_f,m_ and *C*_p,m_ are the concentrations at the membrane surface on the feed and permeate, respectively; *P*_h,f_ is the applied hydraulic pressure on the feed side of the membrane; and the permeate side of the membrane is assumed to be at ambient pressure. Assuming isothermal conditions and applied pressures less than 100 bar, this equation can be further simplified so that the vapor pressure difference and water flux are linearly dependent on hydraulic and osmotic pressures (*22*). All simulations of water flux in this work solved the nonlinear system of equations above and accounted for concentration polarization, heat transport, and temperature polarization; isothermal conditions are only assumed in the above definition to provide simplified explanations. Full descriptions of the calculations for mass transfer resistance, heat transfer, and concentration polarization are given in the Supplementary Materials.

## Supplementary Materials

### This PDF file includes:

Supplementary Text Figs. S1 to S15 Tables S1 to S4 References
