# Supplementary material for: Pressure-driven distillation using air-trapping membranes for fast and selective water purification
Source: Sci Adv. 2023 Jul 14;9(28):eadg6638. doi: 10.1126/sciadv.adg6638 (PMC10348675; doi:10.1126/sciadv.adg6638)
Supplement: Supplementary file 1 — Supplementary Text Figs. S1 to S15 Tables S1 to S4 References [file sciadv.adg6638_sm.pdf]

Science Advances
AAAS

Supplementary Materials for

Pressure-driven distillation using air-trapping membranes for fast and selective water purification

Duong T. Nguyen *et al.*

Corresponding author: Anthony P. Straub, anthony.straub@colorado.edu

Sci. Adv. **9**, eadg6638 (2023)
DOI: 10.1126/sciadv.adg6638

This PDF file includes:

Supplementary Text
Figs. S1 to S15
Tables S1 to S4
References

Supplementary Text

Structure, hydrophobicity, and stability of the fabricated membranes

The structure of the fabricated anodic aluminum oxide (AAO) membranes was analyzed using field emission scanning electron microscopy (FESEM). Pore size and surface porosity of the modified AAO membranes were determined by Image J analysis of FESEM images. Assuming cylindrical pores of AAO membranes, the ratio of nanopore area over the membrane area is considered the membrane porosity. The effective size of the nanopores were considered as the diameter of the circles having the same area as the pore. The equivalent pore size and porosity were then estimated from at least twenty different locations on each sample.

FESEM imaging revealed that deposition of the of the platinum layer, which was used to create the thin hydrophobic layer as outlined in the Materials in Methods, led to minor narrowing of the pore diameter of the membranes from 79.3±12.7 to 75.5±14.9 nm and the surface porosity after modification was found to be 14.5 % (fig. S1D). Membranes with three different pore sizes were tested with diameters of 75.5, 43.3, and 27.1 nm and porosities of 14.5, 15.7, and 13.1%, respectively, as determined by FESEM images. The surface morphology of fabricated AAO membranes appeared to be identical to that of the pristine membranes (fig. S1A,C,D), which is expected with very thin layers of sputter platinum of less than 5 nm and hydrophobic coating of less than 1 nm. Fabricated AAO membranes showed no noticeable change in structure after pressurized desalination at 60 °C for 18 h, after immersion in boiling water for 2 h, or after exposure to 1000 ppm chlorine at pH 4 for 36 h (fig. S1E,F). Contact angles of the dry membranes before testing, after desalination, after chlorine exposure, and after ozone exposure were 162.2±2.0°, 161.4±1.3°, 161.1±1.9°, and 160.1±1.3°, respectively (fig. S2). This implies the hydrophobic coating remains strongly bound to the AAO membranes after prolonged exposure to water at elevated temperature and containing chemical oxidants.

The thickness of the hydrophobic layer was quantified by measuring the depth that the platinum layer entered the pores. FESEM images were taken using a backscattered electron detector after coating the samples with a carbon layer of about 5 nm to avoid charging. The average penetration depth of the platinum layer into the pore was retrieved by Image J based on the high contrast between the elements: platinum is in bright region (atomic weight of 195.1 g/mol) and aluminum oxide is in the dark region (atomic weight of aluminum and oxygen are 27.0 and 16.0 g/mol, respectively) (fig. S3). Estimated thicknesses based on backscattered electron imaging are shown in table S2 and were generally in good agreement with estimations based on the incident angle of platinum sputtering.

Confirmation of surface modification on AAO membranes

We examined the chemical properties of modified AAO membranes by Fourier transform infrared spectroscopy (FTIR). FTIR spectra showed decreased transmittances at 1171 and 1232 cm^{-1} corresponding to $-CF_2$ stretching, indicating that hydrophobic fluorosilane (FAS) molecules were present on the AAO membrane surface (fig. S4). A slight increase in the peak corresponding to hydroxyl groups (-OH) at 3400 cm^{-1} confirmed that substantial consumption of -OH groups occurred. Energy-dispersive X-ray spectroscopy (EDS) analysis showed changes in the elemental composition of pristine and modified AAO membranes. New characteristic F and Si peaks appear at 0.7 and 1.7 keV in the modified samples, indicating 1.57% and 0.37% wt of F and Si on an atomic basis (fig. S5A,B). Along with a high water contact angle, EDS and FTIR spectra provide evidence of the effective hydrophobic modification with low-surface energy FAS. Notably, the bonding between silane molecules and AAO substrate is stable under chlorine exposure (1000 ppm in 36 h at pH 5) as there is no substantial difference between FTIR spectra of the chlorine-exposed and the unused hydrophobic AAO membranes (fig. S4).

Simulating mass and heat flux across membranes

The governing equations for mass flux in the system are provided in the Materials and Methods. Heat flux transferred across the membrane, q, is the sum of convective and conductive heat transfer:

$$q = J_w h_{vap} + \frac{k_m}{\delta}(T_{f,m} - T_{p,m}) \qquad \text{(S1)}$$

where k_m is membrane thermal conductivity, δ is membrane thickness, h_{vap} is the enthalpy of vaporization of water (40.65 kJ mol^{-1}). Convective heat is the heat of vaporization for water transport from the feed to the permeate. Conductive heat is the heat transport from the permeate back to the feed through the membrane material and air gap.

Temperature polarization due to convective heat transfer leads to a lower temperature at the feed-membrane interface and a higher temperature at the permeate-membrane interface, which reduces the overall vapor pressure difference (*34*). Temperature polarization was accounted for using the following equations:

$$T_{f,m} = T_{f,b} - \frac{q}{h_f + J_w c_l} \qquad \text{(S2)}$$

$$T_{p,m} = T_{p,b} + \frac{q}{h_p + J_w c_l} \qquad \text{(S3)}$$

where h_f and h_p are the heat transfer coefficients of the feed and permeate boundary layers, respectively, and c_l is the specific heat capacity of liquid water.

Concentration polarization accounts for the accumulation of rejected solutes near the feed-membrane interface and decreases the water flux due to an elevated osmotic pressure difference

across the membrane. Solute concentration at the membrane-solution interface on the feed side ($C_{f,m}$) is given by (*24*):

$$C_{f,m} = C_{f,b} e^{\frac{J}{k_f}} \quad \text{(S4)}$$

where k_f is the mass transfer coefficient through the feed boundary layer, and $C_{f,b}$ is the bulk salt concentration in the feed. It should be noted that on the permeate, the solute concentration at the membrane surface, $C_{p,m}$, and in the bulk, $C_{p,b}$, are considered equal.

Heat and mass transfer coefficients were assumed to be fixed since the variations in h (500–22000 W m^{-2}K^{-1}) and k (0.02–0.04 kg m^{-2}s^{-1}) result in less than 5% changes in the water flux. Thus, our simulations assume $h_f = h_p = 1000$ W m^{-2}K^{-1} and $k_f = 0.0278$ kg m^{-2}s^{-1}.

The vapor permeability coefficient of water (B_w) was determined via the evaporation rate of water obtained from the Hertz hypothesis and total transport resistance (*24*):

$$B_w = \varepsilon \sqrt{\frac{M_w}{2\pi R_g T}} \left[R_t + R_{i,f} + R_{i,p}\right]^{-1} \quad \text{(S5)}$$

where ε is the membrane porosity, M_w is the molecular weight of water, R_g is the universal gas constant, R_t is the transmission resistance, and $R_{i,f}$ and $R_{i,p}$ are the interfacial resistances at the liquid-vapor interfaces of the feed and permeate, respectively .

Transmission and interfacial resistances can be calculated by the following equations (*24*):

$$R_t = \frac{\left(1 - \frac{p_0(T)}{p_t}\right) v_w \delta}{4 D_{wa}} + \frac{1}{\eta} \quad \text{(S6)}$$

$$R_i = \frac{1 - \sigma(T,P)}{\sigma(T,P)} \quad \text{(S7)}$$

where $p_0(T)$ is the vapor pressure of water at temperature T, p_t is the pressure of the gas mixture in the membrane pores, v_w is the mean molecular speed of water vapor, D_{wa} is the diffusion coefficient of water in air, η is the transmission probability, and $\sigma(T, P)$ is the condensation coefficient of water (also referred to as the mass accommodation coefficient of water).

Transmission probability in cylindrical nanopores can be calculated using Berman's formula (*54*):

$$\eta = 1 + \frac{L^2}{4} - \left(\frac{L}{4}\right)(L^2 + 4)^{\frac{1}{2}} - \frac{\left[(8 - L^2)(L^2 + 4)^{\frac{1}{2}} + (L^3 - 16)^2\right]}{72\, L (L^2 + 4)^{\frac{1}{2}} - 288\, ln\left[L + (L^2 + 4)^{\frac{1}{2}}\right] + 288\, ln 2} \quad \text{(S8)}$$

where $L = \delta/a$ is the pore aspect ratio.

We solved the non-linear system of equations to simulate transmembrane mass and heat flux accounting for the impacts of temperature and concentration polarization. The models above have

been widely used for vapor transport in related membrane processes (e.g., membrane distillation, osmotic distillation) with a high degree of accuracy (*19*, *51*).

Determination of membrane pore wetting pressure and theoretical minimum thickness

Pore wetting should be avoided to maintain the air gap that enables selectivity in the fabricated membranes. To resist wetting from an applied hydraulic pressure, membranes should have a small pore radius and high hydrophobicity as described by the Young-Laplace equation (*22*, *34*):

$$\Delta P_{max} = \left|\frac{2\beta\,\gamma_{LV}\,\cos\theta_{eq}}{r}\right| \quad \text{(S9)}$$

where β is a pore geometry factor, γ_{LV} is the surface tension of the liquid-vapor interface, θ_{eq} is the equilibrium water contact angle, r is the pore radius, and ΔP_{max} is the maximum hydraulic pressure difference across the membrane meniscus prior to wetting, also known as the liquid entry pressure (LEP). The LEP values for various pore sizes and contact angles are presented in fig. S6A. Assuming an equilibrium contact angle of 120°, LEP values for 20, 40, and 80 nm pore size membranes are 72.6, 36.3, and 18.2 bar, respectively, according to the Young-Laplace equation.

To prevent pore wetting, the pore length must also be long enough to prohibit wetting of the pore from being thermodynamically favorable. Assuming the membrane has cylindrical pores, a tortuosity of 1, and a pore length equal to membrane thickness, the critical aspect ratio to prevent pore wetting can be defined as follows (*24*):

$$\frac{\delta}{r} > \frac{2}{3(\cos\theta - \cos\theta_{eq})}\left(\left(\frac{1}{1+sin\theta} + sin\theta\right)\frac{1}{1+sin\theta} + sin\theta\right) \quad \text{(S10)}$$

where δ is the membrane thickness, r is the pore radius, θ is the geometric angle between the pore axis and a tangential line to the liquid-vapor interface. For a given hydraulic pressure difference, we calculate the geometric contact angles as follows:

$$cos\,\theta = -\frac{r\Delta P}{2\gamma_{LV}} \quad \text{(S11)}$$

$$cos\,\theta_{eq} = -\frac{r_{max}\Delta P}{2\gamma_{LV}} \quad \text{(S12)}$$

where r_{max} is the maximum pore radius corresponding to the intrinsic contact angle θ_{eq}(assumed to be 120º).

Equation S10 always results in a smaller pore size than Equation S9 given a finite membrane thickness, therefore it is the more conservative constraint for pore wetting. Minimum thicknesses for relevant ranges of pore sizes and applied pressures are shown in fig. S6B. Using an applied pressure of 6.89 bar and a pore size of 75.5 nm, the membrane thickness should be at least 116 nm to maintain the air gap and avoid wetting. All fabricated membranes presented in this work have hydrophobic layer thicknesses above this threshold. Membrane fabricated with thicknesses below this threshold showed wetting, as predicted from theory.

Accounting for effect of concentration polarization

Concentration polarization was accounted for in measurements by measuring both the pure DI water flux ($J_{w,DI}$) and the water flux with 50 mM NaCl ($J_{w,NaCl}$) at the same applied pressure difference, ΔP. These measurements were used with the water flux equations in both scenarios (*55*):

$$J_{w,DI} = L_p \, \Delta P \qquad \text{(S13)}$$

$$J_{w,NaCl} = L_p(\Delta P - \Delta\pi_m) \qquad \text{(S14)}$$

where L_p is the water permeability coefficient of the membrane and $\Delta\pi_m$ is the osmotic pressure difference between the feed-membrane and permeate-membrane interfaces. From the above equations, $\Delta\pi_m$ can be calculated as follows:

$$\Delta\pi_m = \Delta P(1 - \frac{J_{w,NaCl}}{J_{w,DI}}) \qquad \text{(S15)}$$

The salt concentration at feed-membrane interface ($C_{f,m}$) was then estimated using the Van't Hoff equation(*36*):

$$C_{f,m} = \Delta\pi_m \frac{V_m}{\phi R_g T \nu M} \qquad \text{(S16)}$$

where v is the Van't Hoff coefficient, ϕ is the osmotic coefficient, and M is the molar mass of water. For a monovalent salt such as NaCl, the Van't Hoff coefficient is 2. Note that the above equation assumes that the concentration in the permeate does not contribute substantially to the osmotic pressure difference as the membranes are highly selective for solutes tested in this work. The concentration polarization factor is defined as the ratio of the concentration at the feed-membrane interface ($C_{f,m}$) and the bulk feed concentration ($C_{f,b}$):

$$CP = \frac{C_{f,m}}{C_{f,b}} \qquad \text{(S17)}$$

The apparent salt rejection (R_{app}) and true salt rejection (R_{true}) are calculated as follows:

$$R_{app} = 1 - \frac{C_p}{C_{f,b}} \qquad \text{(S18)}$$

$$R_{true} = 1 - \frac{C_p}{C_{f,m}} \qquad \text{(S19)}$$

where C_p is the permeate concentration.

Performance testing of commercial thin-film composite reverse osmosis membranes

The performance of the fabricated air-trapping membranes was compared to that of thin-film composite (TFC) polyamide RO membranes: SW30-XLE, NF90, and NF270 (Dupont, DE, USA). Polyamide RO membranes were stored in a cold dark room at a temperature of 4 °C to prevent any degradation. To obtain the permeability-selectivity trade-off, SW30-XLE membranes were chlorinated at pH 7 using different chlorine concentrations ranging from 100 to 10000 ppm for 1 h as described in the literature and compared to curves determined in prior work (*56*, *57*).

Agreement between water vapor flux and predictions from the Dusty-Gas Model

Using the average pore size determined from FESEM images, experimental water fluxes of the fabricated AAO membranes can be compared to simulated values using the Dusty-Gas Model (Equations 1-7 and S1–S8). In modeling the water flux of membranes, several assumptions were made regarding membrane properties. Thermal conductivity of AAO membranes was determined using an existing empirical equation (*58*):

$$k_m = 1.32 - \varepsilon \qquad \text{(S20)}$$

where k_m is the thermal conductivity of the membrane ($W\ m^{-1}K^{-1}$) and ε is the membrane porosity (46). The AAO membranes have straight cylindrical pores, and their tortuosity, τ, was approximated to equal 1.

The average pore sizes and porosities of the three AAO membranes tested are estimated as 75.5, 43.3, and 27.1 nm and 14.5, 15.7, and 13.1% from FESEM images. The thickness of the thick hydrophobic membranes is found to be approximately 50 μm using a micrometer. We note that membranes with thick air layers were used to validate transport models since interfacial resistances (which require an empirical condensation coefficient) dominate for the sub-200 nm thick air layers (Fig. 3B).

Experimental water fluxes and predictions based on the Dusty-Gas Model were found to be in good agreement (fig. S7). Experimental water flux measurements from hydrophobic membranes with a thickness of 50 μm and pore sizes of 75.5, 43.3, and 27.1 nm were within 10% of model predictions for applied pressures ranging from 3.45 to 34.5 bar.

Long-term desalination by fabricated AAO membranes

We examined the desalination performance of the uniformly coated AAO membranes with a pore size of 80 nm, a thickness of 50 μm, and a porosity of 15% under an applied hydraulic pressure of 13.8 bar and 60 °C for a period of 7 days (168 hours). Water flux and salt rejection were collected daily and remained identical during continuous operation (fig. S8A). During the seven-day long test, normalized water flux remained between 0.16 and 0.17 kg $m^{-2}h^{-1}$ and salt rejection decreased over time but still maintained above 99.5%. Contact angles of the membranes remained at 162 ± 1° after a week of desalination, reaffirming no substantial loss in hydrophobicity (fig. S8B).

Liquid entry pressure and transport in wetted membranes

We gradually increased the transmembrane pressure difference, and continually monitored water flux and salt rejection. There was a threshold pressure, called the liquid entry pressure (LEP), where water transport transitioned from a salt rejecting desalination regime with nonwetted pores to a non-

salt rejecting regime with wetted pores. The liquid entry pressures (LEPs) for 20, 40, and 80 nm pore size membranes were 48.3, 31.0, and 13.8 bar, respectively, in agreement with Young-Laplace predictions for an intrinsic contact angle of 120° (Equation S9). When the hydraulic pressure on the feed exceeded the liquid entry pressure, membrane wetting occurred and was irreversible as subsequent decreases in hydraulic pressure showed non-selective liquid water transport in the wetted regime.

We studied liquid flow through wetted pores to validate that membrane pores were not clogged during modification. Liquid flow through the pores in the wetted regime was four orders of magnitude higher than vapor transport in desalination regime when the pores were not wetted (fig. S9). Liquid water flux is consistent with laminar liquid flow through a cylindrical pore modeled using the Hagen-Poiseuille equation (*59*):

$$J_{w,l} = \frac{\varepsilon {d_p}^2 \Delta P}{32 \tau \delta \eta_w} \qquad \text{(S23)}$$

where d_p is the membrane pore diameter, ε is the membrane porosity, ΔP is the transmembrane hydraulic pressure, τ is the membrane tortuosity, η_w is the dynamic viscosity of water (8.89×10^{-4} Pa s at 25 °C), δ is the membrane thickness, and $J_{w,l}$ is the liquid water flux through the membrane.

Impact of heat transfer on water vapor transport

Transport of water vapor through the membrane results in heat transfer associated with evaporation and condensation which can negatively impact the membrane flux. We estimated the heat transfer in our experiments and quantified the contribution to flux decline from temperature polarization, a phenomenon that occurs as the liquid-vapor interface on the feed side cools and the interface on the permeate side heats, reducing the partial vapor pressure difference that drives water vapor through the membrane. The impact of temperature polarization can be estimated by balancing heat transfer from the latent heat of vaporization, which is known from the water flux, and conductive heat transfer through the thin air gap (Equation S2, S3). For the ultrathin membranes used in this study, it was found that temperature polarization resulted in a negligible temperature difference across the membrane (less than 1×10^{-3} °C), which would have less than a 2.5% decrease in the water flux. Negative impacts from heat transfer across the membrane were thus concluded to be negligible for our system since the ultrathin membranes had an extremely high thermal conductivity that prevented any substantial buildup of temperature. We note that the experimental measurement of the temperature difference across the membrane as not possible due to the extremely low difference in temperature.

To draw more general conclusions on the effect of heat transfer in the process, simulations of heat transfer were conducted for small membrane elements and large-scale membrane modules.

Membranes with different representative thicknesses and thermal conductivities were simulated using the Dusty-Gas Model accounting for the latent heat of evaporation/condensation and conductive heat transfer across the membrane (Equations 1-7 and S1-S8). The flux decline due to temperature polarization was generally less than 10% in membranes with thicknesses varying from 0.1 to 1 μm that had a high thermal conductivity comparable to the alumina membranes (1.0 W $m^{-1}K^{-1}$). We observed a maximum temperature difference of 2.7×10^{-3} °C at a thickness of 30 μm (fig. S10B, C). Water flux decline due to temperature polarization was as high as 16% for 10 μm thick membranes with a lower thermal conductivity comparable to hydrophobic polymeric membranes (0.1 W $m^{-1}K^{-1}$), which corresponded to a maximum temperature difference of 0.021 °C. The relatively low effect of temperature polarization on water flux is attributed to conductive heat transfer from the permeate to the feed, which prevents a substantial temperature difference from forming across the membrane (fig. S10A).

Experimental measurements supported the minor effect of heat transfer on the water flux. Specifically, measurements of DI water flux through all membranes showed a near linear increase in water flux as a function of the applied pressure (Fig. 2A). If substantial temperature polarization was occurring, we would expect that water flux would have a nonlinear relationship with hydraulic pressure, where high water fluxes at high pressures would lead to substantial convective heat transfer that would limit the achievable water flux.

Module-scale modeling of water vapor transport

Bulk temperature changes in large-scale membrane modules due to heat transfer across the membranes were simulated using the finite-difference method. We modeled a membrane module with co-current flow, which is an approximation of the behavior in a pressurized membrane module. The differential equations for mass and heat transfer were discretized using the finite difference method to obtain the heat flux, flow rates, and concentrations along the module:

$$\frac{dQ_f(A)}{dA} = \frac{dQ_p(A)}{dA} = J_w(T_{f,m}(A_l), T_{p,m}(A_l), C_{f,m}(A_l), C_{p,m}(A_l), \Delta P) \qquad \text{(S24)}$$

$$\frac{d[Q_f(A_l)c_l(T_f(A_l))]}{dA_l} = \frac{d[Q_p(A_l)c_l(T_p(A_l))]}{dA_l} = q(T_{f,m}(A_l), T_{p,m}(A_l), C_{f,m}(A_l), C_{p,m}(A_l), \Delta P) \qquad \text{(S25)}$$

$$\frac{d[Q_f(A_l)C_f(A_l)]}{dA_l} = \frac{d[Q_p(A_l)C_p(A_l)]}{dA_l} = 0 \qquad \text{(S26)}$$

where Equation S24 indicates the water volume balance, Equation S25 expresses the balance of conductive heat and convective heat fluxes, and Equation S26 represents the mass balance of the solute. Q_f and Q_p are the mass flow rates on feed and permeate sides while A_l is the area of the membrane element. The water flux, J_w, and heat flux, q, terms in Equations S24 and S25 were calculated from Equations 7 and S1. The equations were solved by the modified Powell method in

conjunction with the element-scale analysis. The boundary conditions of the solutions were $Q_f(0) = Q_{f,0}$, $Q_p(N) = Q_{p,0}$, $T_f(0) = T_{f,0}$, $T_p(N) = T_{p,0}$, $C_f(0) = C_{f,0}$, and $C_p(N) = C_{p,0}$.

Simulating module-scale changes in bulk temperature due to heat transfer

We conducted simulations to explore whether heat transfer through the membrane via the enthalpy of vaporization could lead to large-scale changes in the bulk feed and permeate solution temperatures. Water and heat fluxes over the entire module length were used to obtain bulk temperatures in a variety of operating conditions and membrane properties (fig. S11A). In fig. S11C, the x-axis is the relative position in the simulated membrane module, and the y-axis is the bulk temperature difference between the feed and permeate sides. Four realistic representative values of membrane thickness and thermal conductivity were used for the model. Simulations for membrane modules with a 50% recovery showed that, for any realistic range of membrane properties, the water flux losses due to bulk temperature changes were less than 2.8% (fig. S11B). The temperature difference was minimal with the thermal conductivity of 1.0 W $m^{-1}K^{-1}$. When the thermal conductivity was 0.1 W $m^{-1}K^{-1}$, the maximum temperature difference was about 0.018 K. Thus, heat transfer across the membrane will result in minor losses in water flux and negligible changes in bulk temperature difference in pressure-driven distillation since heat conduction through the membrane and air gaps alleviate the detrimental effects of latent heat transfer.

Rejection of boron, urea, and *N*-nitrosodimethylamine

Boron rejection was tested using a feed concentration of 5 mg/L which is close to that in real seawater (*60*). Boron concentrations of feed and permeate samples were quantified using inductively coupled plasma mass spectroscopy (Agilent 7700x, CA, USA). The detection limit of the instrument is 0.241 μg/L, allowing precise determination of boron rejection even when it is close to 99%.

Urea rejection was examined using 0.3 M urea as the feed solution (*48*). Urea concentrations in collected samples were measured via colorimetric reaction of urea and diacetyl monoxime. Immediately after collecting feed and permeate samples, diacetyl monoxime (DAM, 50 g/L), thiosemicarbazide (TSC, 2 g/L) and 50% (v/v) H_2SO_4 were prepared as stock solutions. Ferric sulfate ($Fe_2(SO_4)_3$, 600 mg/L) was prepared by dissolving $Fe_2(SO_4)_3$ in 50% (v/v) H_2SO_4. Urea, DAM, and TSC solutions were stored in amber bottles at 4 °C to avoid any photodegradation and were stable for at least a week. To measure urea concentrations, 2 mL of sample was added to 0.1 mL DAM, 17 μL TSC, 17 μL of $Fe_2(SO_4)_3$, and 1.3 mL H_2SO_4. This mixture was heated in a water bath at 85 °C for 30 min in the dark. Absorption spectra were obtained by a UV-Vis spectrophotometer (Hach DR6000, CO, USA) after letting the samples cool to room temperature.

Urea concentrations were analyzed via absorbance at the wavelength of 520 nm. As shown in fig. S13A and S13B, two distinct regions of absorbance were found that correspond to two concentration ranges: 0.001–0.3 mM (purple complex) and greater than 0.3 mM (orange complex). The absorbance increases with urea concentration in the concentration range from 0.001 to 0.3 mM but decreases with urea concentration at concentrations greater than 0.3 mM.

Rejection of NDMA was measured with an initial concentration of 100 mg/L, a temperature of 25 °C and an applied pressure of 10.3 bar. The high feed concentration was used to allow a detection limit of at least 99.9% rejection. NDMA concentration was immediately measured after the rejection tests using a high-performance liquid chromatograph (HPLC) (Agilent 1220, CO, USA) equipped with a UV-Vis detector and a 5 μm particle size reverse phase column (Agilent Eclipse Plus C-18, CO, USA)(*50*). The mobile phase was 10/90 (v/v) methanol/ultrapure water running at a flow rate of 1 mL/min. The wavelength of NDMA detection is 228 nm. Each sample was injected twice to confirm the NDMA measurement was accurate. The retention time and injection volume were 2.6 min and 100 μL, respectively. A calibration curve was built to determine NDMA concentration of the feed and permeate waters within the range of 0.1 to 5000 μM (fig. S13C).

Exposure of the membranes to chemical oxidants

The oxidation tolerance of fabricated AAO and commercial polyamide membranes were examined by submerging in chlorine and ozone solutions at a certain concentration and pH inside a beaker fully covered by aluminum foil to prevent any photodegradation. 1000 ppm chlorine solution was prepared daily and 25 ppm ozone solution was continuously produced from an ozone generator. pH of chlorine and ozone solutions was adjusted using 0.1 M HCl and 0.1 M NaOH. Chlorine and ozone concentrations were monitored throughout the experiment to ensure the membranes were exposed to consistent doses of the oxidants (fig. S14).

Total chlorine concentration was measured by a handheld colorimeter and chlorine test kit (Hach DR300, CO, USA). Since the detection range of this meter is 0.02–2 mg/L, collected chlorine samples were diluted 500 times before measurement. A powder pillow containing *N*,*N*-diethyl-*p*-phenylenediamine was mixed with 10 mL of diluted sample. The mixture was slowly swirled for 20 s to form a pink complex.

Ozone concentration was measured via UV-Vis absorbance at the wavelength of 258 nm (*51*). A 1 cm quartz cuvette was employed, and DI water was used as the baseline for UV-Vis measurements. For each measurement, 1 mL of ozone solution was immediately taken from the reactor, transferred to a clean cuvette, and the corresponding absorbance was measured. The entire procedure was carried out in less than 5 s to prevent loss of ozone due to evaporation.

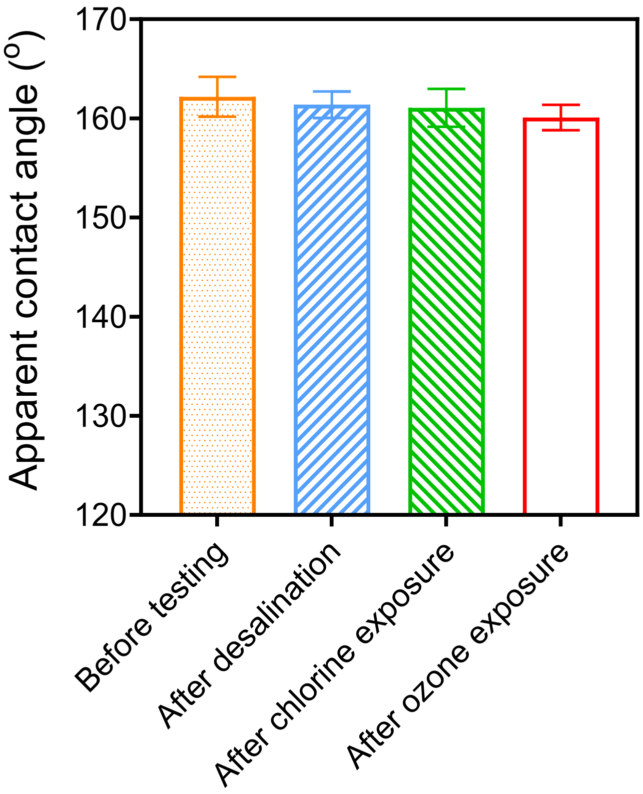

Figure S1 | Water contact angles of fabricated membranes. Static water contact angles of the dry hydrophobic AAO membranes before testing, after desalination, after chlorine exposure, and after ozone exposure.

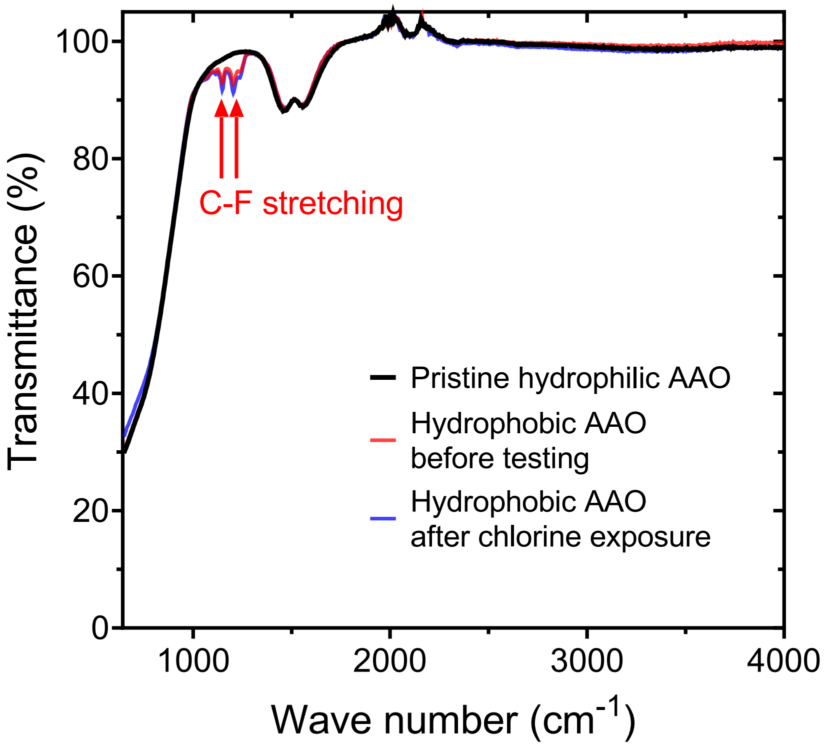

Figure S2 | Fourier transform infrared spectroscopy (FTIR) analysis of fabricated membranes. FTIR spectra of pristine hydrophilic, untested hydrophobic, and chlorine-exposed hydrophobic AAO membranes.

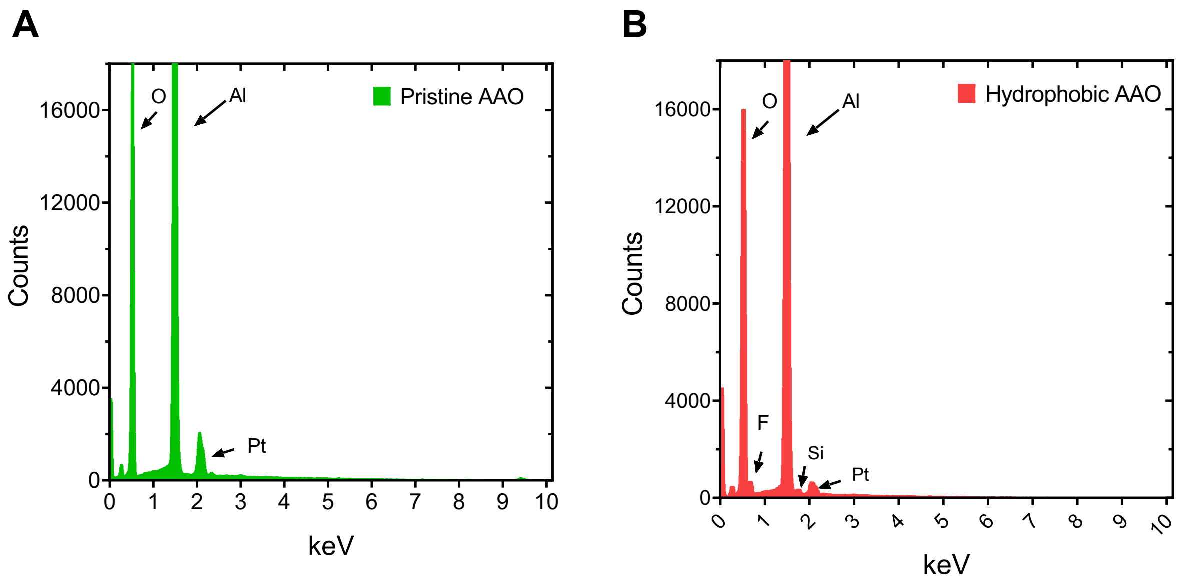

Figure S3 | Energy dispersive X-ray spectroscopy of fabricated membranes. EDS spectra of (**A**) pristine hydrophilic and (**B**) hydrophobic AAO membranes before testing.

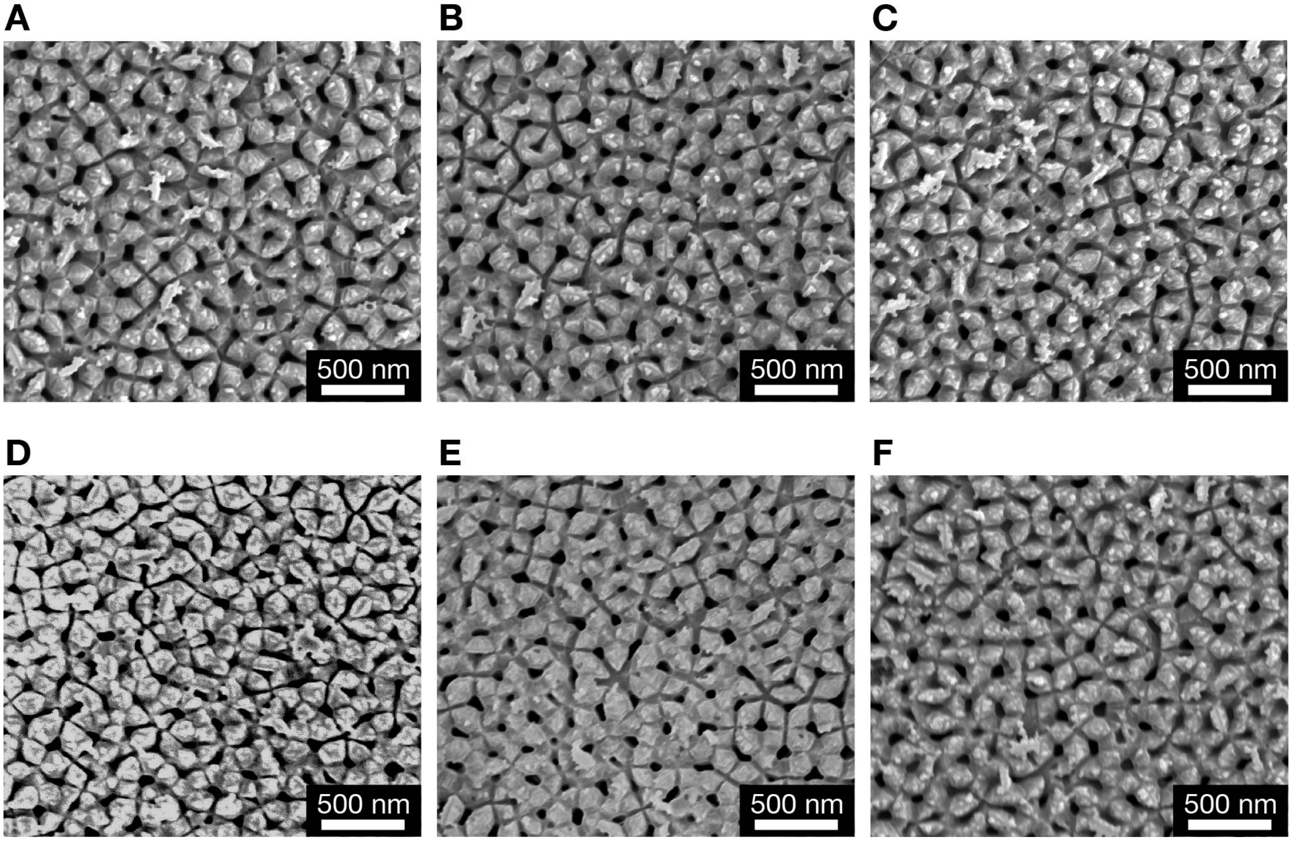

Figure S4 | Scanning electron microscopy images of membrane top surface. SEM images of (**A**) pristine AAO, (**B**) annealed AAO, (**C**) annealed AAO modified with a hydrophobic coating, and (**D**) annealed AAO modified with a thin air layer using a sputtering and hydrophobic modification procedure. (**E**) Hydrophobic AAO after desalination testing and (**F**) hydrophobic AAO after exposure to 1000 ppm chlorine for 36 h at pH 4.

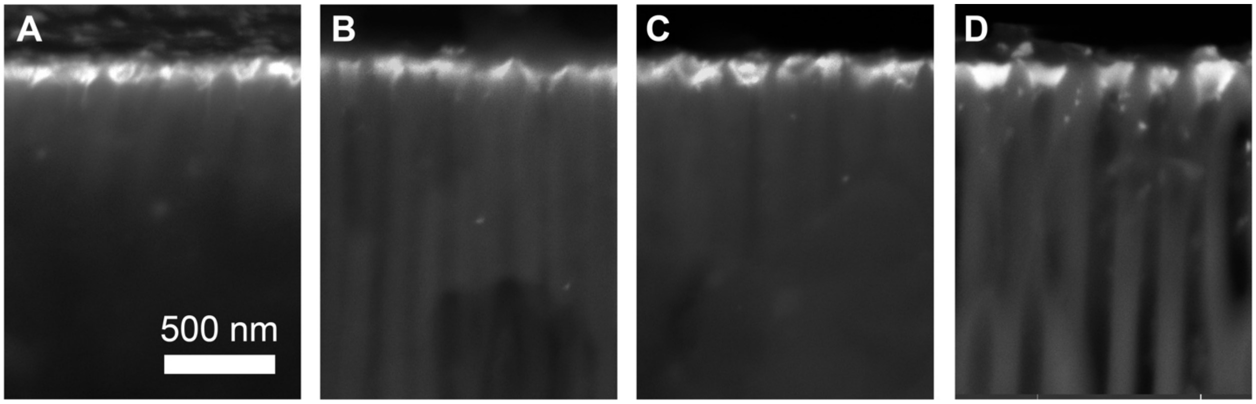

Figure S5 | Scanning electron microscopy backscattering detector images of hydrophobic platinum layer. FESEM images using backscattering electron detector of the membrane cross section. Lighter areas have a higher atomic weight associated with the platinum sputtered layer. Each image corresponds to a different incident sputtering angle, which results in a different penetration depth. Incident angles are (**A**) 55°, (**B**) 60°, (**C**) 65°, and (**D**) 75°.

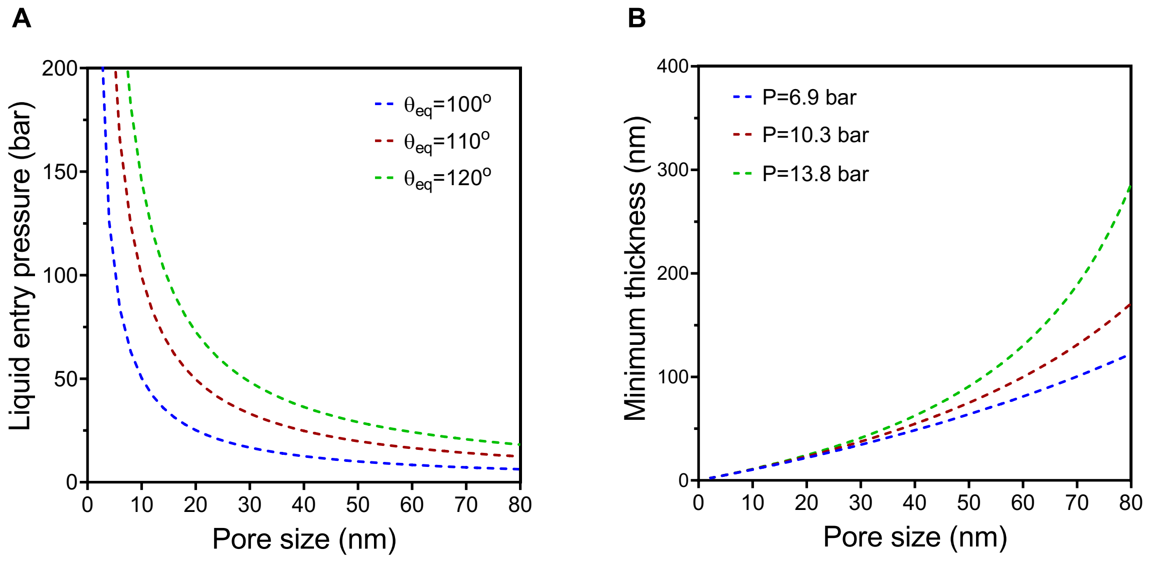

Figure S6 | Simulated liquid entry pressure and minimum possible thickness. (**A**) Modeled liquid entry pressure as a function of pore sizes with different intrinsic contact angles (θ_{eq}), (**B**) Modeled minimum possible air layer thickness as a function of pore size with various applied pressures (assuming θ_{eq} = 120°).

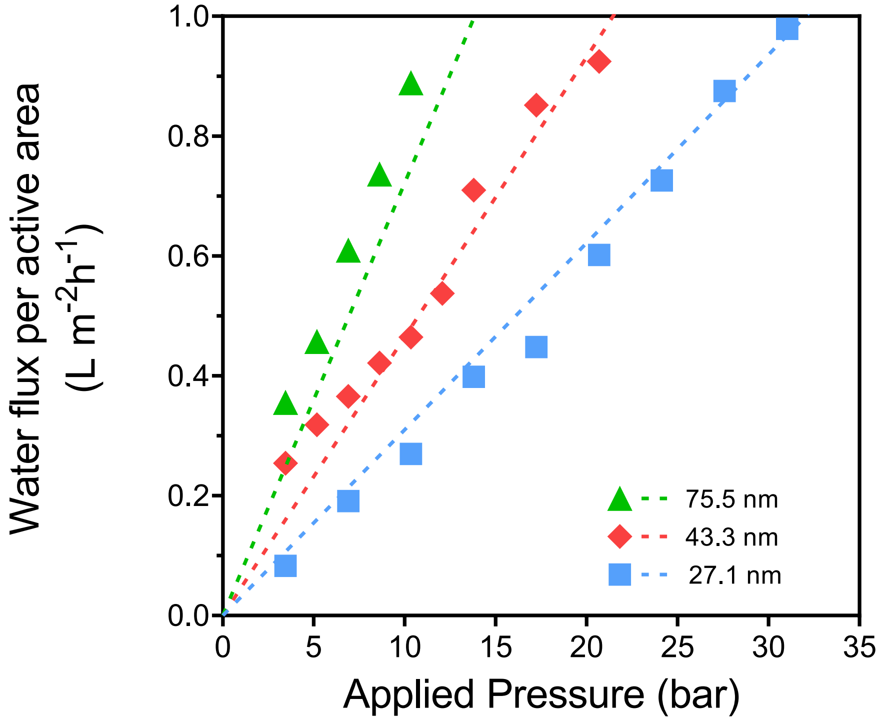

Figure S7 | Experimental and simulated water flux. Comparison of experimental (symbols) and simulated (dotted lines) water vapor fluxes using fabricated AAO membranes with pore sizes of 75.5, 43.3, and 27.1 nm and the same thickness of 50 µm under varying hydraulic pressures of 3.45 to 34.5 bar. Water flux is normalized to active pore area.

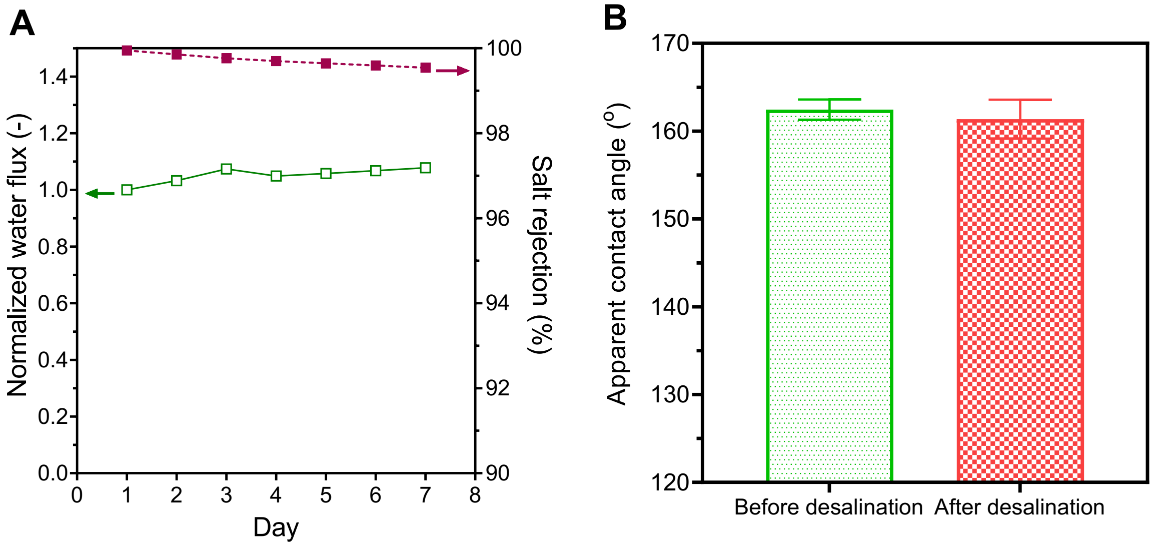

Figure S8 | Long-term desalination testing. (**A**) Normalized water flux and salt rejection of the fabricated AAO membrane over one week of desalination. (**B**) Apparent contact angle recorded before testing and after 1 week of operation.

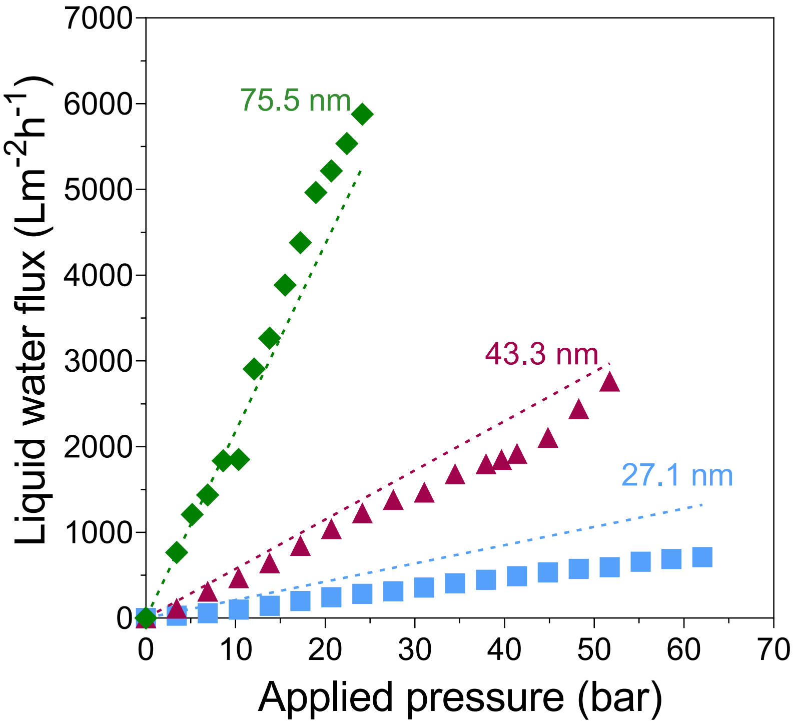

Figure S9 | Liquid water flux through fully wetted pores. Measured liquid water flux versus hydraulic pressure when the membrane pores are fully wetted with water. Dotted lines show modeled liquid water flux by Hagen-Poiseuille equation.

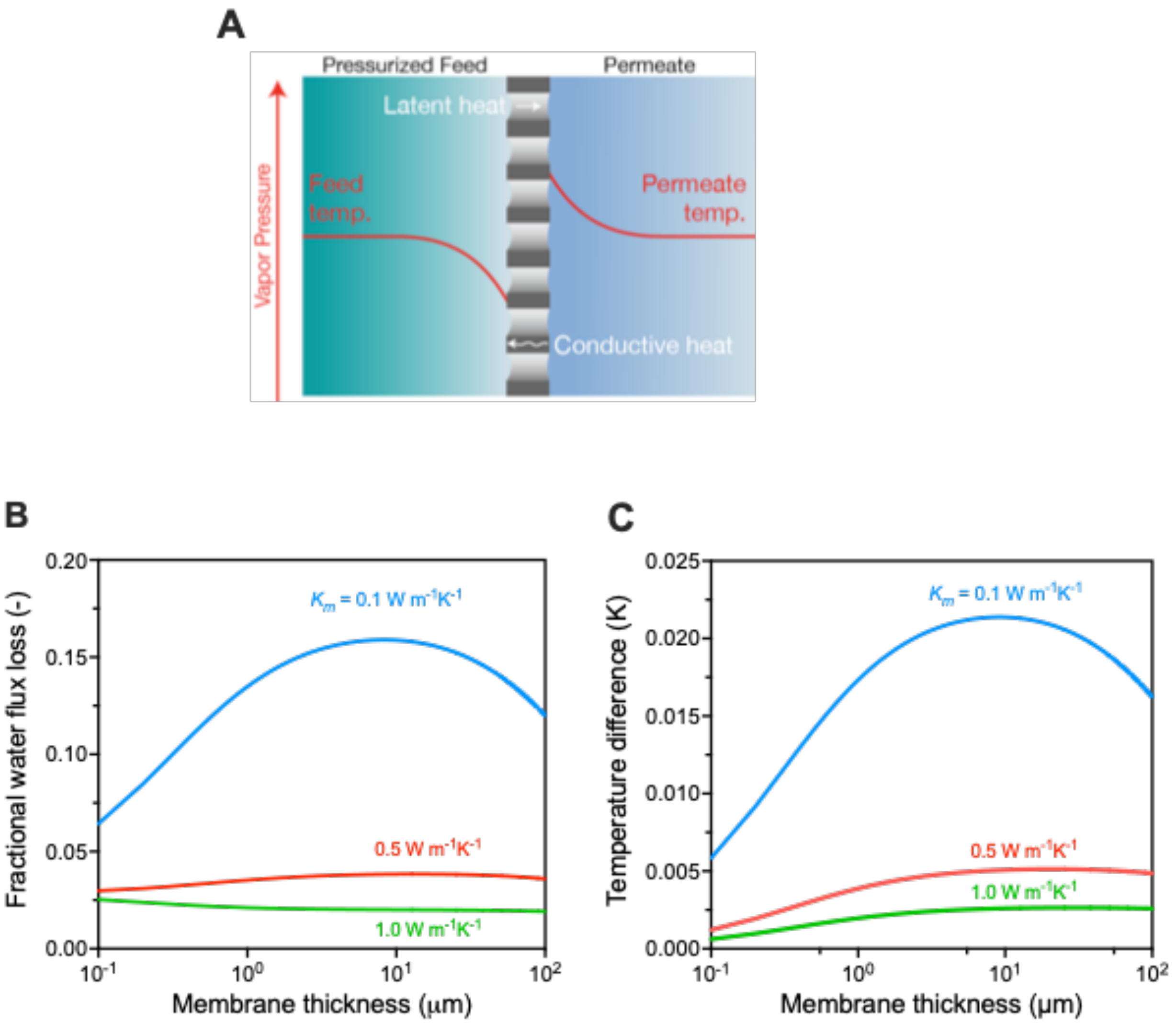

Figure S10 | Effect of heat transfer on water flux. (**A**) Schematic diagram of latent and conductive heat transfer across the coupon-scale membrane and temperature polarization on the feed and permeate side of the membrane. **(B)** Fractional water losses caused by temperature polarization for membranes with varying thickness and thermal conductivity, K_m. **(C)** Temperature differences formed across vapor-gap RO membrane due to the transfer of latent and conductive heat. All simulations assume an initial temperature of 25 °C in the feed and permeate streams, an applied pressure of 13.8 bar, a pore size of 80 nm, and a porosity of 15%, and unless otherwise indicated.

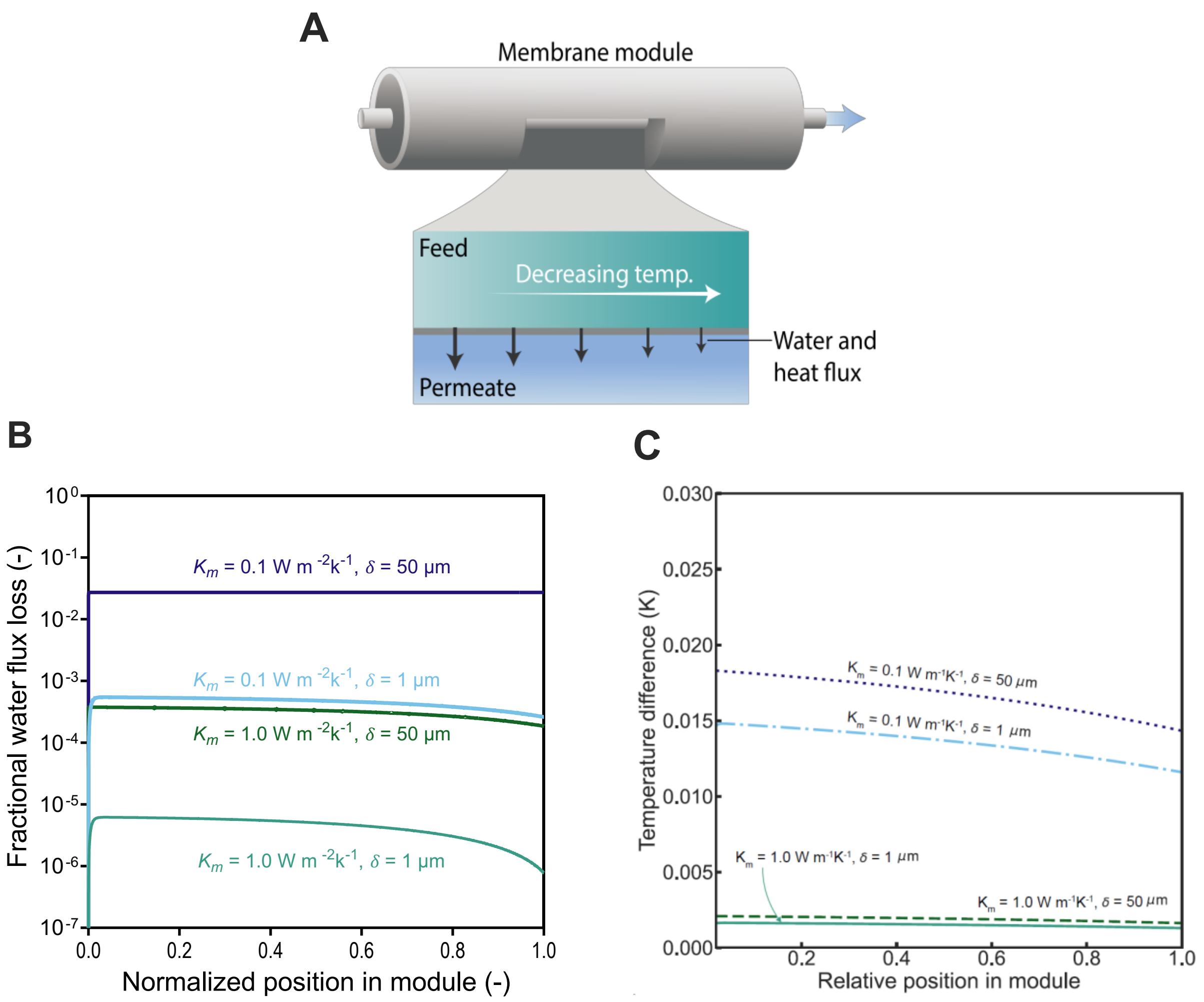

Figure S11 | Effect of heat transfer on module-scale performance. **(A)** Schematic diagram of heat accumulation in a membrane module where water vapor transport causes a decrease in the bulk temperature of the feed and an increase in the bulk temperature of the permeate. **(B)** Fractional water flux loss due to bulk temperature differences across the membrane for module scale system operating with a 50% recovery of a 50 mM sodium chloride feed solution. **(C)** Simulated temperature difference between the feed and permeate along the length of the membrane module. All simulations assume an initial temperature of 25 °C in the feed and permeate streams, an applied pressure of 13.8 bar, a pore size of 80 nm, and a porosity of 15%, and unless otherwise indicated. The feed and permeate flow rates were 20 kg s^{-1} and their recoveries were set at 50%.

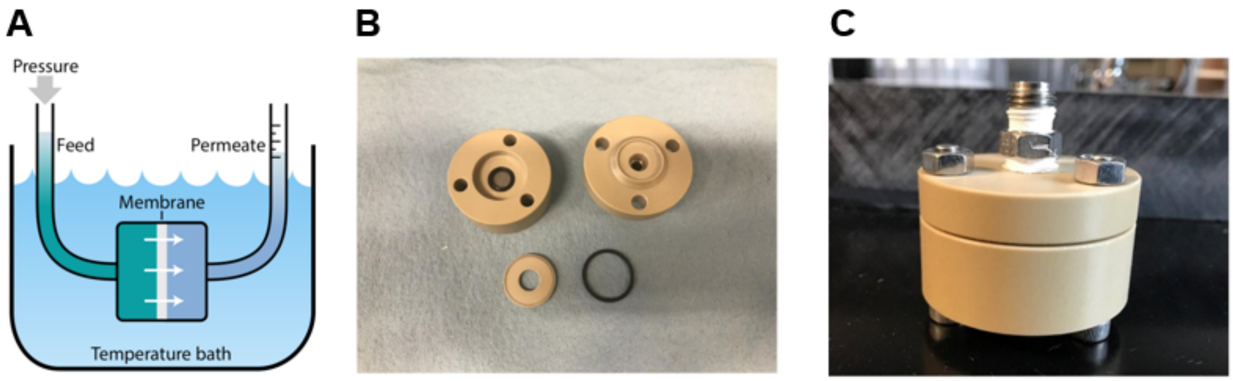

Figure S12 | Desalination testing apparatus. (**A**) Schematic diagram of the testing apparatus where the hydrophobic membrane is sealed in a flow cell and hydraulic pressure is applied to the feed side. (**B, C**) Coupon-scale membrane flow cell with a membrane area of 0.86 cm^2.

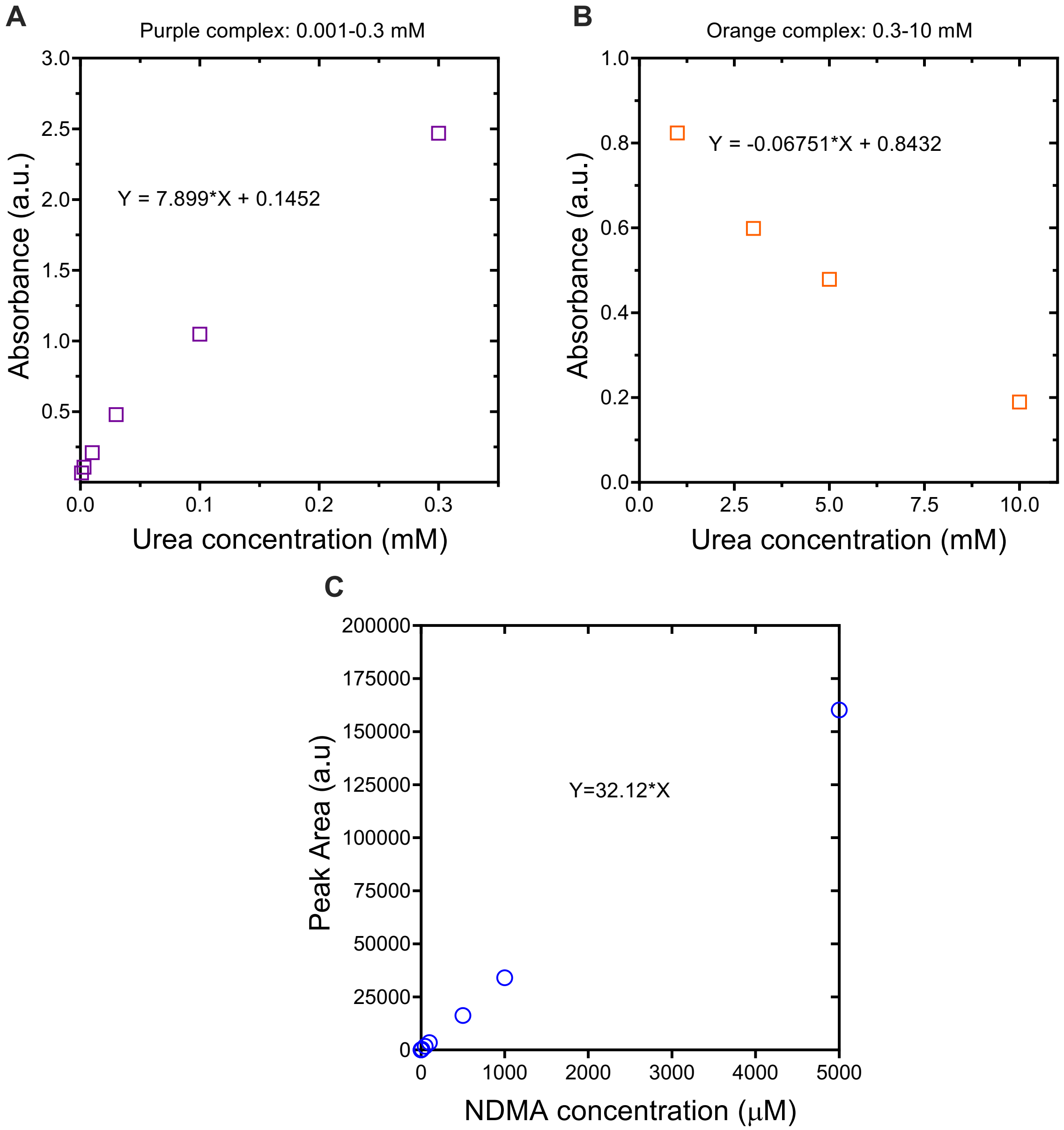

Figure S13 | Urea and NDMA calibration curves. Urea calibration curve with concentration ranges of (**A**) 0.001–0.3 mM and (**B**) 0.31–10 mM. (**C**) NDMA calibration curve with concentrations ranging from 0.1 to 5000 μM.

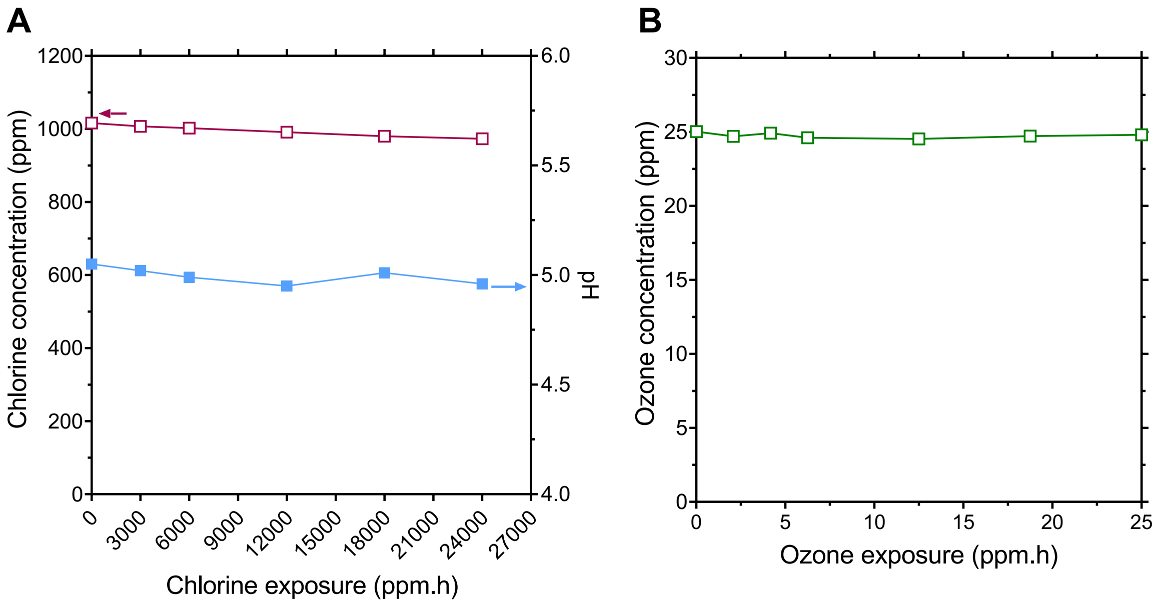

Figure S14 | Chlorine and ozone concentrations during oxidant exposure tests. Concentration profiles of (**A**) chlorine and (**B**) ozone during oxidant exposure experiments at pH 4 and 7.1, respectively.

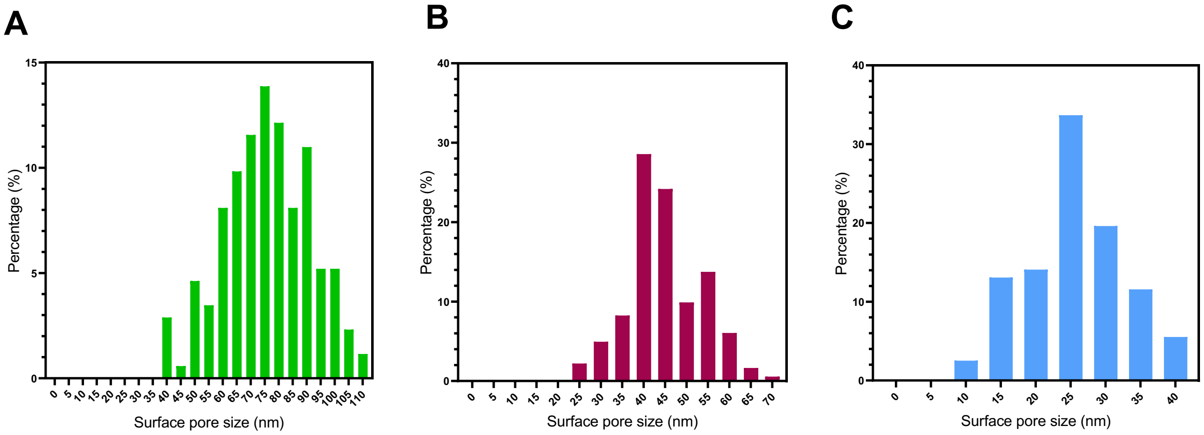

Figure S15 | Pore size distribution extracted from SEM imaging. Pore size distributions of the fabricated membranes based on alumina substrates in this work. The pore sizes of the substrates claimed by from the manufacturers (InRedox) are (A) 80 nm, (B) 40 nm, and (C) 20 nm.

Table S1 | Liquid entry pressure values reported in the literature. Measured liquid entry pressures of commercial membranes made of polytetrafluoroethylene (PTFE), polyvinylidene fluoride (PVDF), polypropylene (PP), and polyethylene (PE) and fabricated membranes made of carbon nanotubes, nanofibrous polystyrene (PS), and poly(ethylene chlorotrifluoroethylene) (ECTFE) shown with their corresponding pore sizes and water contact angles.

Membranes	Measured liquid entry pressure (bar)	Pore size (nm)	Contact angle (º)	References
Polytetrafluoroethylene (PTFE)	2.82	200	NA	(22)
Polyvinylidene fluoride (PVDF)	2.00<br>2.04	300<br>265	139º<br>NA	(22, 23)
Polypropylene (PP)	2.50	220	93.5º	(22)
Polyethylene (PE)	0.70<br>0.60	210<br>106	108.3º<br>106.4º	(24)
Carbon nanotubes	0.55	27.3	113.3º	(25)
Nanofibrous polystyrene (PS)	1.5	190	111º	(26)
Poly(ethylene chlorotrifluoroethylene) (ECTFE)	2.7	260	93.4º	(27)

Table S2 | Thickness of the hydrophobic layer. Estimated thickness of platinum selective layer on aluminum oxide substrate with an average pore size of 75.5 nm from backscattering images and pore geometry.

Incident angles	55°	60°	65°	75°
Estimated thickness based on backscattering SEM images (nm)	119.0±12.7	135.5±19.4	161.2±20.3	189.3±34.8
Estimated thickness based on pore geometry (nm)	107.8	130.8	161.9	281.8

Table S3 | Water permeability values normalized to active membrane area and total membrane area. Pure water permeabilities of the fabricated alumina membranes with and without normalization by porosity. The membranes with an average pore size of 75.5 nm, an average porosity of 14.5%. and the thickness varying from 100 µm to 119 nm. Feed water is DI water, temperature is 60 ºC, and hydraulic pressure is 6.89 bar.

Thickness of hydrophobic layer (nm)	Water permeability normalized by active area (kg $m^{-2}h^{-1}bar^{-1}$)	Water permeability normalized by true surface area (kg $m^{-2}h^{-1}bar^{-1}$)
119	8.87	1.29
136	8.38	1.22
161	7.56	1.10
189	6.32	0.916
20,000	0.28	0.041
50,000	0.13	0.019
100,000	0.05	0.007

Table S4 | Desalination process comparison. Comparison of three desalination processes: reverse osmosis, membrane distillation, and pressure-driven distillation.

	Reverse osmosis	Membrane distillation	Pressure-driven distillation
Driving force	Hydraulic pressure difference (*2*)	Partial vapor pressure gradient induced by temperature difference (Antoine equation) (*61*)	Partial vapor pressure gradient induced by hydraulic pressure difference (Kelvin equation) (*22*)
Transport mechanism and resistances	Solution-diffusion transport through polymer matrix (*7*)	Vapor transport in Knudsen and molecular diffusion regimes (*61*)	Vapor transport in Knudsen diffusion regime. Interfacial resistances from evaporation and condensation are important (*36*)
Ideal membrane properties	Thin layer of dense polyamide or cellulose acetate (*2*)	Pore size of 100–1000 nm. Optimal thickness of hydrophobic layer is 50–200 µm (*61*)	Pore size less than 100 nm. Thin hydrophobic layer less than 1 µm
Estimated energy efficiency	1–2 kWh m^{-3} (*2*)	Greater than 7.7 kWh m^{-3} (*19*)	Similar to RO (*)
Oxidation resistance	Polyamide membranes tolerate chloramines but degrade from chlorine and ozone (*62*)	Hydrophobic materials generally tolerant to chlorine and ozone (*17*, *41*)	Hydrophobic materials generally tolerant to chlorine and ozone (*17*, *41*)
Selectivity	Governed mostly by size, charge, and dielectric constant of the solutes (*63*)	Controlled by volatility or Henry's constant of the solutes (*64*)	Controlled by volatility or Henry's constant of the solutes (*64*)

Nomenclature

Latin letters

A_l = area of the membrane element

B_w = vapor permeability coefficient of water

c_l = specific heat capacity of liquid water

$C_{f,b}$ = bulk salt concentrations on the feed

$C_{f,m}$ = salt concentrations at the feed-membrane interface

$C_{p,b}$ = bulk salt concentrations on the permeate

d_p = membrane pore diameter

$D_{s,a}$ = Knudsen diffusion coefficient

$D_{s,m}$ = molecular diffusion coefficient

D_{wa} = diffusion coefficient of water vapor in air

h = heat transfer coefficient

h_{vap} = enthalpy of vaporization

J_w = water vapor flux through the membrane

$J_{w,l}$ = liquid water flux through the membrane

k = mass transfer coefficient

k_g = thermal conductivity of the gas

k_m = membrane thermal conductivity

k_f = mass transfer coefficients through feed boundary layer

k_s = thermal conductivity of the solid phase

l = optical path length

L = pore aspect ratio

L_p = water permeability coefficient of the membrane

M = molar mass of water

M_w = molecular weight of solute

n = number of molecules in system

P_v = partial vapor pressure

Pv_0 = saturated vapor pressure

p_t = partial pressure of gas mixtures inside the pores

P = hydraulic pressure

$P_{v,s}$ = vapor pressure of the solute

ΔP = transmembrane hydraulic pressure

$\Delta P_{\max}$ = liquid entry pressure

q = transmembrane heat flux

Q_f = flow rate on feed side

Q_p = flow rate on permeate side

r = membrane pore radius

r_{max} = membrane pore radius corresponding to the intrinsic contact angle

R_{app} = apparent solute rejection

R_c = radius of curvature.

R_g = universal gas constant

$R_{i,f}$ = interfacial resistances at liquid/vapor interface of the feed

$R_{i,p}$ = interfacial resistances at liquid/vapor interface of the permeate

R_t = transmission resistance

R_{true} = true solute rejection

$T_{f,b}$ = bulk feed temperature

$T_{f,m}$ = temperature at the feed-membrane interface

$T_{p,b}$ = permeate feed temperature

$T_{p,m}$ = temperatures at the permeate-membrane interface

v_w = mean velocity of water vapor

V_m = molar volume of liquid water

Greek letters

α = absorbance at 258 nm

β = pore geometry factor

γ_{LV} = surface tension of the liquid-vapor interface

γ_s = activity coefficient

δ = membrane thickness

ε = membrane porosity

η = transmission probability

η_w = dynamic viscosity of water

θ = measured water contact angle

θ_{eq} = equilibrium water contact angle

ϕ = osmotic coefficient

π = osmotic pressure

σ = condensation coefficient

τ = membrane tortuosity

ν = Van't Hoff coefficient

ω = molar absorptivity

REFERENCES AND NOTES

1. A. A. Uliana, N. T. Bui, J. Kamcev, M. K. Taylor, J. J. Urban, J. R. Long, Ion-capture electrodialysis using multifunctional adsorptive membranes. *Science* **372**, 296–299 (2021).

2. M. Elimelech, W. A. Phillip, The future of seawater desalination: Energy, technology, and the environment. *Science* **333**, 712–717 (2011).

3. D. Hou, T. Li, X. Chen, S. He, J. Dai, S. A. Mofid, D. Hou, A. Iddya, D. Jassby, R. Yang, L. Hu, Z. J. Ren, Hydrophobic nanostructured wood membrane for thermally efficient distillation. *Sci. Adv.* **5**, eaaw3203 (2019).

4. K. Zuo, W. Wang, A. Deshmukh, S. Jia, H. Guo, R. Xin, M. Elimelech, P. M. Ajayan, J. Lou, Q. Li, Multifunctional nanocoated membranes for high-rate electrothermal desalination of hypersaline waters. *Nat. Nanotechnol.* **15**, 1025–1032 (2020).

5. J. R. Werber, C. O. Osuji, M. Elimelech, Materials for next-generation desalination and water purification membranes. *Nat. Rev. Mater.* **1**, 16018 (2016).

6. S. Ling, Z. Qin, W. Huang, S. Cao, D. L. Kaplan, M. J. Buehler, Design and function of biomimetic multilayer water purification membranes. *Sci. Adv.* **3**, e1601939 (2017).

7. H. B. Park, J. Kamcev, L. M. Robeson, M. Elimelech, B. D. Freeman, Maximizing the right stuff: The trade-off between membrane permeability and selectivity. *Science* **356**, eaab0530 (2017).

8. T. E. Culp, B. Khara, K. P. Brickey, M. Geitner, T. J. Zimudzi, J. D. Wilbur, S. D. Jons, A. Roy, M. Paul, B. Ganapathysubramanian, A. L. Zydney, M. Kumar, E. D. Gomez, Nanoscale control of internal inhomogeneity enhances water transport in desalination membranes. *Science* **371**, 72–75 (2021).

9. M. R. Chowdhury, J. Steffes, B. D. Huey, J. R. McCutcheon, 3D printed polyamide membranes for desalination. *Science* **361**, 682–686 (2018).

10. Y. Wen, R. Dai, X. Li, X. Zhang, X. Cao, Z. Wu, S. Lin, C. Y. Tang, Z. Wang, Metal-organic framework enables ultraselective polyamide membrane for desalination and water reuse. *Sci. Adv.* **8**, eabm4149 (2022).

11. M. Di Vincenzo, A. Tiraferri, V.-E. Musteata, S. Chisca, R. Sougrat, L.-B. Huang, S. P. Nunes, M. Barboiu, Biomimetic artificial water channel membranes for enhanced desalination. *Nat. Nanotechnol.* **16**, 190–196 (2021).

12. Y. Yao, P. Zhang, C. Jiang, R. M. DuChanois, X. Zhang, M. Elimelech, High performance polyester reverse osmosis desalination membrane with chlorine resistance. *Nat. Sustain.* **4**, 138–146 (2021).

13. K. L. Cho, A. J. Hill, F. Caruso, S. E. Kentish, Chlorine resistant glutaraldehyde crosslinked polyelectrolyte multilayer membranes for desalination. *Adv. Mater.* **27**, 2791–2796 (2015).

14. S. Zhao, C. Jiang, J. Fan, S. Hong, P. Mei, R. Yao, Y. Liu, S. Zhang, H. Li, H. Zhang, C. Sun, Z. Guo, P. Shao, Y. Zhu, J. Zhang, L. Guo, Y. Ma, J. Zhang, X. Feng, F. Wang, H. Wu, B. Wang, Hydrophilicity gradient in covalent organic frameworks for membrane distillation. *Nat. Mater.* **20**, 1551–1558 (2021).

15. A. V. Dudchenko, C. Chen, A. Cardenas, J. Rolf, D. Jassby, Frequency-dependent stability of CNT Joule heaters in ionizable media and desalination processes. *Nat. Nanotechnol.* **12**, 557–563 (2017).

16. M. Wang, P. Zhang, X. Liang, J. Zhao, Y. Liu, Y. Cao, H. Wang, Y. Chen, Z. Zhang, F. Pan, Z. Zhang, Z. Jiang, Ultrafast seawater desalination with covalent organic framework membranes. *Nat. Sustain.* **5**, 518–526 (2022).

17. Y. Zhang, P. Zhao, J. Li, D. Hou, J. Wang, H. Liu, A hybrid process combining homogeneous catalytic ozonation and membrane distillation for wastewater treatment. *Chemosphere* **160**, 134–140 (2016).

18. Z. Wang, T. Horseman, A. P. Straub, N. Y. Yip, D. Li, M. Elimelech, S. Lin, Pathways and challenges for efficient solar-thermal desalination. *Sci. Adv.* **5**, eaax0763 (2019).

19. A. Deshmukh, C. Boo, V. Karanikola, S. Lin, A. P. Straub, T. Tong, D. M. Warsinger, M. Elimelech, Membrane distillation at the water-energy nexus: Limits, opportunities, and challenges. *Energ. Environ. Sci.* **11**, 1177–1196 (2018).

20. W. Liu, R. Wang, A. P. Straub, S. Lin, Membrane design criteria and practical viability of pressure-driven distillation. *Environ. Sci. Technol.* **57**, 2129–2137 (2023).

21. K. P. Lopez, R. Wang, E. A. Hjelvik, S. Lin, A. P. Straub, Toward a universal framework for evaluating transport resistances and driving forces in membrane-based desalination processes. *Sci. Adv.* **9**, eade0413 (2023).

22. J. Lee, R. Karnik, Desalination of water by vapor-phase transport through hydrophobic nanopores. *J. Appl. Phys.* **108**, 044315 (2010).

23. M. Khayet, Membranes and theoretical modeling of membrane distillation: A review. *Adv. Colloid Interface Sci.* **164**, 56–88 (2011).

24. A. P. Straub, N. Y. Yip, S. Lin, J. Lee, M. Elimelech, Harvesting low-grade heat energy using thermo-osmotic vapour transport through nanoporous membranes. *Nat. Energy* **1**, 16090 (2016).

25. O. Labban, C. Liu, T. H. Chong, J. H. Lienhard, Relating transport modeling to nanofiltration membrane fabrication: Navigating the permeability-selectivity trade-off in desalination pretreatment. *J. Membr. Sci.* **554**, 26–38 (2018).

26. G. M. Geise, H. B. Park, A. C. Sagle, B. D. Freeman, J. E. McGrath, Water permeability and water/salt selectivity tradeoff in polymers for desalination. *J. Membr. Sci.* **369**, 130–138 (2011).

27. J. R. Werber, A. Deshmukh, M. Elimelech, The critical need for increased selectivity, not increased water permeability, for desalination membranes. *Environ. Sci. Technol. Lett.* **3**, 112–120 (2016).

28. A. Deshmukh, J. Lee, Membrane desalination performance governed by molecular reflection at the liquid-vapor interface. *Int. J. Heat Mass Transf.* **140**, 1006–1022 (2019).

29. F. Restagno, L. Bocquet, T. Biben, Metastability and nucleation in capillary condensation. *Phys. Rev. Lett.* **84**, 2433–2436 (2000).

30. J. Lee, T. Laoui, R. Karnik, Nanofluidic transport governed by the liquid/vapour interface. *Nat. Nanotechnol.* **9**, 317–323 (2014).

31. P. M. Winkler, A. Vrtala, P. E. Wagner, M. Kulmala, K. E. J. Lehtinen, T. Vesala, Mass and thermal accommodation during gas-liquid condensation of water. *Phys. Rev. Lett.* **93**, 075701 (2004).

32. W. Chen, S. Chen, T. Liang, Q. Zhang, Z. Fan, H. Yin, K.-W. Huang, X. Zhang, Z. Lai, P. Sheng, High-flux water desalination with interfacial salt sieving effect in nanoporous carbon composite membranes. *Nat. Nanotechnol.* **13**, 345–350 (2018).

33. H. Hyung, J.-H. Kim, A mechanistic study on boron rejection by sea water reverse osmosis membranes. *J. Membr. Sci.* **286**, 269–278 (2006).

34. M. T. Pickett, L. B. Roberson, J. L. Calabria, T. J. Bullard, G. Turner, D. H. Yeh, Regenerative water purification for space applications: Needs, challenges, and technologies towards 'closing the loop'. *Life Sci. Space Res.* **24**, 64–82 (2020).

35. T. Merle, W. Pronk, U. von Gunten, MEMBRO$_3$X, a novel combination of a membrane contactor with advanced oxidation (O_3/H_2O_2) for simultaneous micropollutant abatement and bromate minimization. *Environ. Sci. Technol. Lett.* **4**, 180–185 (2017).

36. S. Ling, Z. Qin, C. Li, W. Huang, D. L. Kaplan, M. J. Buehler, Polymorphic regenerated silk fibers assembled through bioinspired spinning. *Nat. Commun.* **8**, 1387 (2017).

37. Y.-H. Chuang, A. Y.-C. Lin, X.-H. Wang, H. Tung, The contribution of dissolved organic nitrogen and chloramines to nitrogenous disinfection byproduct formation from natural organic matter. *Water Res.* **47**, 1308–1316 (2013).

38. W. Wang, X. Du, H. Vahabi, S. Zhao, Y. Yin, A. K. Kota, T. Tong, Trade-off in membrane distillation with monolithic omniphobic membranes. *Nat. Commun.* **10**, 3220 (2019).

39. Z. Wang, S. Lin, Membrane fouling and wetting in membrane distillation and their mitigation by novel membranes with special wettability. *Water Res.* **112**, 38–47 (2017).

40. F. Zhao, X. Zhou, Y. Shi, X. Qian, M. Alexander, X. Zhao, S. Mendez, R. Yang, L. Qu, G. Yu, Highly efficient solar vapour generation via hierarchically nanostructured gels. *Nat. Nanotechnol.* **13**, 489–495 (2018).

41. P. L. Barclay, J. R. Lukes, Curvature dependence of the mass accommodation coefficient. *Langmuir* **35**, 6196–6202 (2019).

42. L. Chen, J. Ma, Y. Huang, M. Dai, X. Li, Optimization of a colorimetric method to determine trace urea in seawater. *Limnol. Oceanogr. Methods.* **13**, 303–311 (2015).

43. R. Bernstein, S. Belfer, V. Freger, Toward improved boron removal in ro by membrane modification: Feasibility and challenges. *Environ. Sci. Technol.* **45**, 3613–3620 (2011).

44. M. E. Huang, S. Huang, D. L. McCurry, Re-examining the role of dichloramine in high-yield *N*-nitrosodimethylamine formation from *N,N*-dimethyl-α-arylamines, *Environ. Sci. Technol. Lett.* **5**, 154–159 (2018).

45. J. Lee, A. P. Straub, M. Elimelech, Vapor-gap membranes for highly selective osmotically driven desalination. *J. Membr. Sci.* **555**, 407–417 (2018).

46. G. Vaartstra, Z. Lu, J. H. Lienhard, E. N. Wang, Revisiting the schrage equation for kinetically limited evaporation and condensation. *J. Heat Transfer* **144**, 080802 (2022).

47. I. W. Eames, N. J. Marr, H. Sabir, The evaporation coefficient of water: A review. *Int. J. Heat Mass Transf.* **40**, 2963–2973 (1997).

48. L. Eykens, K. De Sitter, C. Dotremont, L. Pinoy, B. Van der Bruggen, Characterization and performance evaluation of commercially available hydrophobic membranes for direct contact membrane distillation. *Desalination* **392**, 63–73 (2016).

49. M. Khayet, A. O. Imdakm, T. Matsuura, Monte Carlo simulation and experimental heat and mass transfer in direct contact membrane distillation. *Int. J. Heat Mass Transf.* **53**, 1249–1259 (2010).

50. J. Zuo, S. Bonyadi, T.-S. Chung, Exploring the potential of commercial polyethylene membranes for desalination by membrane distillation. *J. Membr. Sci.* **497**, 239–247 (2016).

51. L. F. Dumée, K. Sears, J. Schütz, N. Finn, C. Huynh, S. Hawkins, M. Duke, S. Gray, Characterization and evaluation of carbon nanotube Bucky-Paper membranes for direct contact membrane distillation. *J. Membr. Sci.* **351**, 36–43 (2010).

52. H. Ke, E. Feldman, P. Guzman, J. Cole, Q. Wei, B. Chu, A. Alkhudhiri, R. Alrasheed, B. S. Hsiao,

Electrospun polystyrene nanofibrous membranes for direct contact membrane distillation. *J. Membr. Sci.* **515**, 86–97 (2016).

53. J. Pan, C. Xiao, Q. Huang, H. Liu, J. Hu, ECTFE porous membranes with conveniently controlled microstructures for vacuum membrane distillation. *J. Mater. Chem. A* **3**, 23549–23559 (2015).

54. A. S. Berman, Free molecule transmission probabilities. *J. Appl. Phys.* **36**, 3356 (1965).

55. Z. Yang, H. Guo, C. Y. Tang, The upper bound of thin-film composite (TFC) polyamide membranes for desalination. *J. Membr. Sci.* **590**, 117297 (2019).

56. X. Chen, C. Boo, N. Y. Yip, Influence of solute molecular diameter on permeability-selectivity tradeoff of thin-film composite polyamide membranes in aqueous separations. *Water Res.* **201**, 117311 (2021).

57. N. Y. Yip, M. Elimelech, Performance limiting effects in power generation from salinity gradients by pressure retarded osmosis. *Environ. Sci. Technol.* **45**, 10273–10282 (2011).

58. B. Abad, J. Maiz, M. Martin-Gonzalez, Rules to determine thermal conductivity and density of anodic aluminum oxide (AAO) membranes. *J. Phys. Chem. C* **120**, 5361–5370 (2016).

59. Y. Chung, D. Park, H. Kim, S.-E. Nam, S. Kang, Novel method for the facile control of molecular weight cut-off (MWCO) of ceramic membranes. *Water Res.* **215**, 118268 (2022).

60. B. Jung, C. Y. Kim, S. Jiao, U. Rao, A. V. Dudchenko, J. Tester, D. Jassby, Enhancing boron rejection on electrically conducting reverse osmosis membranes through local electrochemical pH modification. *Desalination* **476**, 114212 (2020).

61. A. Alkhudhiri, N. Darwish, N. Hilal, Membrane distillation: A comprehensive review. *Desalination* **287**, 2–18 (2012).

62. R. Verbeke, V. Gómez, I. F. J. Vankelecom, Chlorine-resistance of reverse osmosis (RO) polyamide membranes. *Prog. Polym. Sci.* **72**, 1–15 (2017).

63. R. Wang, S. Lin, Pore model for nanofiltration: History, theoretical framework, key predictions,

limitations, and prospects. *J. Membr. Sci.* **620**, 118809 (2021).

64. S. Lee, A. P. Straub, Analysis of volatile and semivolatile organic compound transport in membrane distillation modules. *ACS EST Eng.* **2**, 1188–1199 (2022).
